# Ultrasound-Targeted Nanobubbles Codelivering NKP-1339 and miR-142-5p for Synergistic Mitochondrial Immunogenic Cell Death and PD-L1 Inhibition in Cancer Therapy

**DOI:** 10.34133/bmr.0232

**Published:** 2025-08-08

**Authors:** Yafei Zhang, Chaoqi Liu, Shuai Jin, Liangyun Xie, Qianwen Xiao, Jun Yao

**Affiliations:** ^1^ The First Affiliated Hospital , and College of Clinical Medicine of Henan University of Science and Technology, Luoyang 471003, China.; ^2^ Hubei Key Laboratory of Tumor Microenvironment and Immunotherapy, China Three Gorges University, Yichang, Hubei 443002, China.; ^3^ Medical College of China Three Gorges University, Yichang, Hubei 443002, China.; ^4^ The First Clinical Medical College of Three Gorges University, Center People’s Hospital of Yichang, Yichang, Hubei 443008, China.

## Abstract

The combination of chemical immunotherapy and gene therapy holds great promise for malignant tumor treatment. Here, we developed an ultrasound-targeted liposome nanobubbles system (NKP-1339/miR-142-NBs) for precise codelivery of drugs and genes to treat esophageal squamous cell carcinoma (ESCC) with ultrasound-targeted microbubble destruction (UTMD). This study systematically investigated the system’s therapeutic mechanisms—including mitochondrial dysfunction induction, immunogenic cell death (ICD), and antitumor immune activation—alongside its pharmacokinetics and targeting efficiency. In an ESCC mouse model, NKP-1339/miR-142-NBs combined with ultrasound markedly suppressed tumor growth (79.72% ± 0.1% vs. NB control 18.79% ± 1.29%) through NKP-1339 triggering ICD and miR-142-5p down-regulating programmed death-ligand 1 (PD-L1) expression, synergistically potentiating immune responses. Furthermore, we found that triggering ICD, including the exposure of calreticulin on the cell membrane, was related to altering mitochondrial fission dynamics in the ESCC cells. The down-regulation of PD-L1 expression by miR-142-5p reactivated CD8^+^ T cells by relieving programmed death-1 (PD-1)/PD-L1-mediated immunosuppression, enhancing immune memory and antitumor efficacy. Moreover, the UTMD technique enhanced the tumoral accumulation and penetration of nanobubbles, improving delivery specificity and minimizing off-target effects. This combined treatment strategy, including UTMD, provides a promising translational potential for ESCC therapy.

## Introduction

Esophageal squamous cell carcinoma is the sixth-leading cause of cancer-related deaths and was responsible for 540,000 deaths in 2020 [1]. Despite the effectiveness of traditional treatments including surgery, radiotherapy, and chemotherapy, which induce apoptosis or necrosis in cancer cells through cytotoxic agents [2], these approaches are insufficient to prevent cancer recurrence [3]. Therefore, the development of novel and effective treatment strategies for esophageal cancer has become a critical issue requiring urgent attention.

Recently, immunotherapy is an effective treatment approach that activates the immune system to eliminate tumor cells, thereby preventing tumor recurrence and metastasis. However, immunotherapy faces numerous challenges in clinical oncology, including low immunogenicity and immunosuppressive tumor microenvironment (TME) [4]. Key features of this environment include exhausted cytotoxic T lymphocytes (CTLs), myeloid-derived suppressor cells, tumor-associated macrophages, and regulatory T cells [5]. Induction of immunogenic cell death (ICD) has gained considerable attention in cancer therapy. ICD is a form of immune stimulation-regulated cell death, in which dying cells serve as anticancer immunogens to activate immune responses [6]. It is one of the strategies used in anticancer immunotherapy and is capable of reversing the immunosuppressive TME, thus enhancing sensitivity to immunotherapy. Tumor cells undergoing ICD express tumor-associated antigens and damage-associated molecular patterns (DAMPs), such as surface exposure of calreticulin (CRT) (“eat me” signal), release of high-mobility group box 1 (HMGB1) (“danger” signal), and secretion of adenosine triphosphate (ATP) (“find me” signal). These ICD-associated DAMPs are captured and processed by antigen-presenting cells, and their interaction with dendritic cells (DCs) promotes the maturation and activation of antigen-specific immune responses [7]. This results in the activation of CTL-mediated immune responses, leading to the elimination of tumor cells. Therefore, the development of a safe and effective ICD induction strategy has become a promising and feasible approach for tumor immunotherapy, including the treatment of esophageal cancer.

Traditionally, cytotoxic chemotherapy has been associated with immunosuppressive effects such as those induced by platinum-based compounds [8]. However, clinical studies have demonstrated that a combination of chemotherapy and immunotherapy often produces synergistic effects. Ruthenium-based compounds have emerged as a promising candidate for cancer research owing to their unique mechanisms of action. Among these, NKP-1339, a ruthenium-based complex, sodium trans-[tetrachloridobis (1H-indazole)ruthenate-(III)] (also known as IT-139, KP1339, or BOLD-100), is a novel ICD inducer that has shown significant preclinical activity in various tumor models and has been well tolerated in clinical trials. NKP-1339 induces endoplasmic reticulum (ER) stress and tumor cell death by down-regulating GRP78 (BiP) and disrupting ER homeostasis [9]. Although some chemotherapy drugs such as doxorubicin, mitoxantrone, and platinum-based compounds can also induce ICD [10], the clinical availability of ICD inducers remains limited. Therefore, exploring the immunogenic potential of clinically used anticancer drugs and novel compounds is of great therapeutic significance. However, the targeted nature and nonspecific distribution of monotherapy limit its clinical efficacy [11]. Developing precise delivery platforms to deliver ICD inducers and achieve synergistic effects can markedly improve the therapeutic efficacy of NKP-1339, thereby amplifying the ICD effects and providing a new strategy for cancer immunotherapy.

Immune checkpoint blockade therapy is widely used in clinical practice, and one of its key targets is programmed death-ligand 1 (PD-L1). PD-L1 is highly expressed in most esophageal cancers and correlates with tumor invasiveness, overall survival, and postoperative recurrence, making it an important therapeutic target and potential biomarker. MicroRNA (miRNA) is a noncoding RNA that plays a crucial role in regulating gene expression within the tumor immune microenvironment [12]. miR-142-5p, a tumor-suppressive miRNA, targets PD-L1 to modulate the immune microenvironment and enhances cisplatin-induced apoptosis in ovarian cancer cells [13]. Additionally, miR-142-5p exerts anticancer effects by targeting PIK3CA in non-small cell lung cancer [14], and inhibits esophageal cancer progression by regulating epithelial–mesenchymal transition and stemness [15]. However, as miRNAs are nucleic acid-based drugs with short half-lives in vivo, there is a pressing need for efficient drug delivery systems to enhance their therapeutic efficacy.

Lipid microbubbles (MBs), used as drug and gene delivery carriers, effectively transport therapeutic molecules to tumor tissues via ultrasound (US)-targeted MB destruction, considerably enhancing their therapeutic effects [16]. However, traditional MBs, which typically range in size from to 1 to 4 μm, exhibit limited penetration within tumor tissues. Here, to overcome this limitation, we designed lipid NBs with a size range of 100 to 500 nm, enabling enhanced permeability and retention (EPR) effects that allow passive accumulation within tumor tissues, thereby considerably improving drug delivery efficiency.

**Fig. 1.**
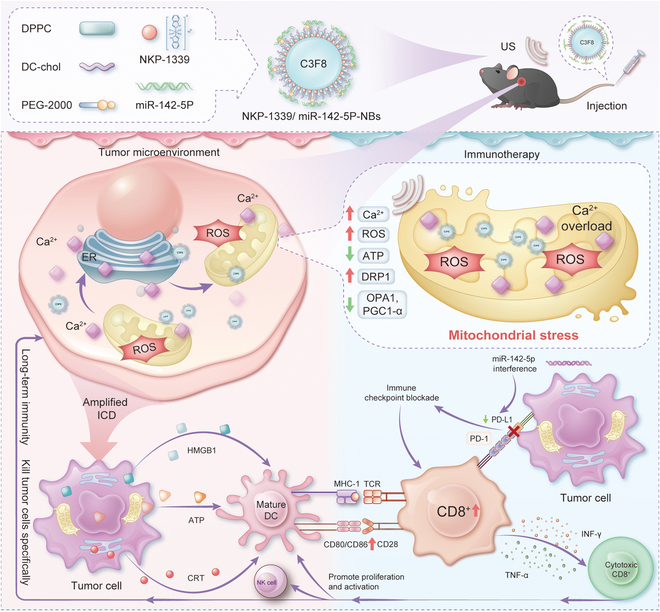
Schematic representation of the design and mechanism of NKP-1339/miR-142-5p-NBs. The nanobubble system enables US-triggered codelivery of NKP-1339 and miR-142-5p to tumor cells. NKP-1339 induces ICD by disrupting mitochondrial function, elevating ROS, and triggering CRT externalization and HMGB1/ATP release. Concurrently, miR-142-5p down-regulates PD-L1 expression, reversing immune suppression. These combined effects facilitate CD8^+^ T cell activation and potentiate antitumor immune responses in esophageal squamous cell carcinoma. DRP1, dynamin-related protein 1; OPA1, optic atrophy 1; PGC1-α, peroxisome proliferator-activated receptor gamma coactivator 1-alpha; MHC I, major histocompatibility complex I; TCR, T cell receptor; CD80, cluster of differentiation 80; CD86, cluster of differentiation 86; CD28, cluster of differentiation 28; PD-1, programmed cell death protein 1; Cytotoxic CD8^+^, cytotoxic CD8^+^ T cells; IFN-γ, interferon gamma; TNF-α, tumor necrosis factor-alpha.

Therefore, we developed a combined strategy for immunogenic chemotherapy and gene therapy using US nanolipid NBs (NKP-1339/miR-142-5P-NBs). This novel drug delivery system offers high biocompatibility and controllability, markedly enhancing tumor tissue permeability and drug targeting via US-triggered cavitation, thereby facilitating efficient drug delivery and precise drug release (Fig. 1). Compared with conventional drug delivery methods, this system markedly increases drug concentrations at the tumor site while reducing systemic toxicity [17]. In vivo studies have shown that this mitochondrial stress-based immunostimulatory strategy can modulate immune cell activity, activating both the innate and adaptive immune systems, thereby reversing the immunosuppressive TME and markedly improving antitumor efficacy in esophageal cancer subcutaneous models.

## Materials and Methods

### Materials and reagents

The following materials and reagents were used: 1,2-Dipalmitoyl-sn-glycero-3-phosphocholine (DPPC; Avanti Polar Lipids, USA); polyethylene glycol 2000 (PEG2000; Avanti Polar Lipids, USA); 3β-[N-(N′,N′-dimethylaminoethane)-carbamoyl]cholesterol (DC-chol; Avanti Polar Lipids, USA); NKP-1339 (MedChemExpress USA; 197723-00-5); Lipomaster 2000 (Vazyme, TL201); TUNEL Assay Kit (Hoffman-LaRoche Ltd, Basel, Switzerland); Fluo-4 AM Dye (Thermo Fisher Scientific, USA); DCFH-DA Dye (Beyotime, China); Annexin V-FITC (fluorescein isothiocyanate)/PI (propidium iodide) Double Staining Kit (BD Biosciences, USA); and MitoTracker® Red (USA).

### Experimental methods

#### Cell and animal models

Male C57BL/6 mice (6 to 8 weeks,18 to 20 g) were purchased from the Wuhan Institute of Biological Products. All mice were housed under specific pathogen-free (SPF) conditions at the Animal Experiment Center of China Three Gorges University. Animal experiments were approved by the Animal Experiment Committee of China Three Gorges University (ethical approval number: 2023120A) and were conducted following the guidelines of the Ministry of Science and Technology of the People’s Republic of China. The mouse esophageal squamous carcinoma Mec25 cell line was provided by the School of Medicine, Shenzhen University, and the International Cancer Center. The culture medium consisted of Dulbecco’s Modified Eagle Medium (DMEM) (Gibco, USA) supplemented with 10% fetal bovine serum (Gibco, USA) and 1% penicillin/streptomycin (Solarbio, Beijing, China), and cells were cultured at 37 °C with 5% CO₂.

### Preparation and characterization of ultrasound nanobubbles

Different types of nanobubbles (NBs) were prepared using the thin-film hydration and mechanical agitation methods. Empty NBs were prepared by mixing DPPC, DSPE-PEG2000, and DC-chol in a 5:2:0.5 mass ratio and dissolved in chloroform and rotary-evaporated at 42 °C for 1 h to form a lipid film. Phosphate-buffered saline (PBS)/glycerol (9:1) was then added, and the mixture was dissolved at 45 °C. The resulting suspension was transferred into a vial, vacuumed, and filled with C_3_F_8_ gas, and mechanically agitated for 90 s to yield a milky suspension of empty NBs. NKP-1339-NBs were prepared by adding NKP-1339 (mass ratio 5:2:0.5:0.25) to the above procedure. For miR-142-5p-NBs, the DC-chol ratio was increased to 5:2:2 to create cationic NBs, which were then incubated with miR-142-5p plasmid (1 μg: 25 μl) for 30 min. NKP-1339/miR-142-NBs were prepared by combining NKP-1339-NBs with the plasmid using the same method. All NBs were sterilized and stored at 4 °C for later use.

The morphology and structure of the prepared NKP-1339/miR-142-NBs were observed using transmission electron microscopy (TEM; Tecnai G2, Thermo Fisher Scientific, USA), and the particle size, distribution, and zeta potential were measured using a Zetasizer Nano ZS to evaluate dispersion and stability.

### Drug encapsulation efficiency of ultrasound NBs

The drug encapsulation efficiency of NKP-1339/miR-142-NBs was determined by ultraviolet–visible (UV–Vis) spectrophotometry and dialysis. Samples were placed in dialysis bags (Union Carbide, USA) and dialyzed in PBS for 6 h, with PBS being replaced every hour to remove free NKP-1339. After dialysis, the samples were mixed with methanol to release NKP-1339, and the absorbance at the maximum absorption wavelength of NKP-1339 (OD value) was measured. The NKP-1339 concentration was calculated using a standard curve. Encapsulation efficiency was calculated using the formula: Encapsulation efficiency (%) = (amount of NKP-1339 loaded/total NKP-1339 mass) × 100%. To verify the loading capacity of miR-142 plasmid, agarose gel electrophoresis was performed.

### Establishment of animal models

For the esophageal cancer xenograft model, mice received subcutaneous injections on the right flank with a suspension containing approximately 1 × 10^8^ Mec25 cells. Once the tumor volume reached 100 mm^3^, the mice were randomly divided into 8 groups: (a) Control group (saline); (b) N-NBs+US group (blank NBs+US); (c) NKP-1339 group (NKP-1339 solution); (d) NKP-1339+US group (NKP-1339+US); (e) NKP-1339-NBs+US group (NKP-1339-loaded NBs+US); (f) miR-142-5p group (gene therapy); (g) miR-142-NBs+US group (miR-142-loaded NBs+US); and (h) NKP-1339/miR-142-NBs+US group (NKP-1339/miR-142 combined-loaded NBs+US). Mice received intravenous injections of the respective solution or NBs suspension every other day, for a total of 6 treatments. For the US groups, US treatment was administered immediately after the injection at the tumor site (1 MHz frequency, 1.5 W/cm^2^ intensity, 50% duty cycle, 120 s). Tumor length (*L*) and width (*W*) were measured before each treatment, and tumor volume (*V*) was calculated using the formula *V* = (*A* × *B*^2^)/2. Mouse body weight changes were recorded throughout the study. Upon completion of treatment, mice were euthanized, and spleen, major organs (heart, liver, spleen, lungs, and kidneys), and tumor samples were collected. Tumor weight was measured to calculate the tumor inhibition rate, and histological analyses were performed. All animal experiments were conducted with *n* = 6 mice per group.

### Pharmacokinetic analysis of ultrasound NBs+US

Tumor-bearing mice were divided into 3 groups: Free Dir, Dir-NBs, and Dir-NKP-1339/miR-142-NBs (3 mice per group). After intravenous injection of 200 μl of the respective solution, fluorescence images were captured at 0, 0.5, 1, 6, 12, and 24 h using the in vivo imaging system (IVIS) system (PerkinElmer, USA) with excitation at 754 nm and emission at 778 nm. Under anesthesia, whole-body fluorescence distribution was monitored to analyze the drug distribution and signal accumulation at the tumor site. Mice were euthanized at 24 h, and tumors and organs were collected to observe fluorescence signal distribution. Semiquantitative analysis was performed using ImageJ software to calculate the fluorescence intensity in tumors and organs, assessing the aggregation efficiency and distribution of Dir-NKP-1339/miR-142-NBs. Tumor-bearing mice were randomly assigned to 3 treatment groups: NBs, NKP-1339-NBs, and NKP-1339/miR-142-NBs. Each group received a 200-μl intravenous injection containing approximately 1 × 10^9^ NBs. Real-time tumor-targeted contrast enhancement was monitored using contrast-enhanced ultrasound (CEUS) imaging (Mindray RESONA7S, equipped with a 14-MHz linear transducer). Imaging data were recorded at 0, 10, 45, 90, and 150 s postinjection. Mice were anesthetized with 2% isoflurane and maintained at a constant body temperature throughout the procedure. Quantitative analysis of signal intensity and decay kinetics was performed using Sonamath software to assess the targeting efficiency of each formulation.

### H&E staining

The tumor tissues and various organs of the mice were embedded and fixed in paraffin, and tissue sections were prepared. After staining with hematoxylin and eosin (H&E), morphological changes in the tumor tissues and organs were observed.

### Immunohistochemistry

After dewaxing and rehydration of the tumor sections (4 μm), antigen retrieval was performed using citrate buffer. Endogenous peroxidase activity was blocked with 3% H₂O₂ solution, followed by blocking with 5% bovine serum albumin (BSA) for 1 h. The sections were incubated overnight at 4 °C with primary antibodies: Bax (1:200, Santa Cruz, sc-20067) and Bcl-2 (1:200, Santa Cruz, sc-7382). After washing with PBS, the sections were incubated with a secondary antibody (goat anti-rabbit IgG, 1:500, Jackson ImmunoResearch) at room temperature for 1 h. Following DAB staining, nuclei were counterstained, and sections were mounted. Images were captured using an optical microscope, and semiquantitative analysis was performed using ImageJ software.

### Immunofluorescence

Tumor sections (4 μm) were dewaxed, rehydrated, and subjected to antigen retrieval, washed, and blocked before incubation with primary antibodies for 12 to 16 h: anti-PD-L1 (1:200, Proteintech, 66248-1-lg), anti-CRT (1:200, Proteintech, 27298-1-AP), anti-CD3 (1:400, Proteintech, 60181-1-Ig), anti-CD8 (1:400, Proteintech, 66868-1-Ig), anti-IFN-γ (1:200, Proteintech, 15365-1-AP), anti-CD80 (1:500, Proteintech, 66406-1-Ig), and anti-CD86 (1:100, Abcam, ab239075). After PBS washing, sections were incubated with fluorescence-conjugated secondary antibodies (1:500, Jackson ImmunoResearch) for 1 h. Cell apoptosis was detected using a TUNEL kit (Hoffman-LaRoche Ltd, Basel, Switzerland), and nuclei were stained with DAPI (4′,6-diamidino-2-phenylindole) before mounting. Images were observed using a laser confocal microscope, and semiquantitative analysis of fluorescence intensity was performed using ImageJ software.

### Western blotting

Total proteins were extracted from tumor tissues or cells using radioimmunopreciptation assay lysis buffer (Solarbio, China) and protein concentration was determined using a bicinchoninic acidkit (Servicebio, China). Proteins were mixed with loading buffer, denatured by boiling at 95 °C for 10 min, and separated by sodium dodecyl sulfate–polyacrylamide gel electrophoresis (10% separating gel). The proteins were transferred to a polyvinylidene difluoride membrane (Millipore, USA). The membrane was blocked with 5% skimmed milk for 1 h and incubated with primary antibodies overnight at 4 °C: PD-L1 (1:5,000, Proteintech, 66248-1-lg), CRT (1:1,000, Proteintech, 27298-1-AP), HMGB1 (1:3,000, Proteintech, 66525-1-Ig), heat shock protein 70 (HSP70) (1:10,000, Proteintech, 10995-1-AP), heat shock protein 90 (HSP90) (1:8,000, Proteintech, 13171-1-AP), DRP1 (1:5,000, Proteintech, 12957-1-AP), OPA1 (1:500, Santa Cruz, sc-393296), PGC1-α (1:2,500, ABclonal, A20995), cyclophilin B (1:1,000, Proteintech, 11607-1-AP), and β-actin (1:1,000, Proteintech, 60008-1-Ig). After washing 3 times with TBST, the membrane was incubated with horseradish peroxidase-conjugated secondary antibody (1:5,000, Proteintech) for 1 h at room temperature and washed 3 times. Protein bands were detected using ECL chemiluminescent reagent (Pierce, USA), and signals were captured using a Bio-Rad gel imaging system (Bio-Rad, USA). Semiquantitative analysis was performed using ImageJ software.

### Reverse transcription-quantitative PCR

Total RNA was extracted from fresh tumor tissues using the Trizol reagent (TaKaRa, Japan) and reverse transcribed to cDNA using the PrimeScript RT kit (TaKaRa, Japan). Reverse transcription-quantitative polymerase chain reaction (RT-qPCR) was performed using SYBR Premix Ex Taq II (TaKaRa, Japan) on an Applied Biosystems 7900HT system with the following thermal cycling parameters: 95 °C for 30 s, followed by 40 cycles of 95 °C for 5 s and 60 °C for 30 s. Actin and U6 were used as reference genes, and relative expression levels were calculated using the 2^−ΔΔCt^ method. The experiment was performed in triplicate to ensure reliability, and primer sequences are provided in Table S1.

### miRNA transfection

A total of 1.5 × 10^5^ Mec25 esophageal cancer cells were seeded into 6-well plates and cultured until they adhered to the surface. The transfection conditions for the miRNA were optimized based on the manufacturer’s instructions and preliminary experiments. A total of 25 μl of Opti-MEM medium was added to 2 tubes: one containing 1.5 μl of Lipomaster 2000 (Vazyme, TL201) and the other containing 0.5 μg of the corresponding miRNA plasmid (GV268, GV268-miR-142-5p, miR mimic NC, miR-142-5p mimic, miR inhibitor NC, and miR-142-5p inhibitor). After mixing the components, the solution was allowed to stand for 5 min to form the transfection complex. The culture medium was removed, and the cells were washed twice with PBS. The complex was then added, and the mixture was gently mixed. After 6 h of transfection, the medium was replaced, and the cells were incubated for an additional 24 to 48 h before being collected for subsequent experiments.

### ROS and mitochondrial detection

A total of 1 × 10^4^ cells were seeded into 24-well plates and incubated overnight at 37 °C with 5% CO₂. The following day, drugs were added, and cells were subjected to US irradiation (1.5 MHz, 45 s, and 50% duty cycle). After incubation for 5 h, the cells were washed 3 times with PBS. To detect reactive oxygen species (ROS), 200 μl of 10 μmol/l DCFH-DA fluorescence probe (Beyotime, Shanghai) was added, and the cells were incubated in the dark for 30 min. After washing with PBS, 500 μl of serum-free DMEM was added, and fluorescence images were acquired using an inverted fluorescence microscope (excitation wavelength, 488 nm; emission wavelength, 525 nm). ImageJ software was used for semiquantitative analysis. In addition, mitochondria were stained with MitoTracker Red (final concentration, 50 to 200 nM, Invitrogen, USA), and after washing with PBS, the dye working solution was added and incubated at 37 °C in the dark for 15 to 30 min. Following 3 PBS washes to remove excess dye, fluorescence signals were detected using a laser scanning confocal microscope (Nikon Eclipse Ti, Japan), and fluorescence intensity and mitochondrial morphology were analyzed.

### Flow cytometry

Mec25 cells were seeded in 6-well plates and subjected to different treatments for 48 h. The following assays were performed: Calcium ion detection: Cells were stained with Fluo-4 AM dye (final concentration, 5 μM) and incubated at 37 °C in the dark for 30 min. After 3 washes with PBS, single-cell suspensions were prepared, and calcium ion fluorescence intensity was measured using a flow cytometer (BD FACSVerse, USA). ROS detection: Cells were stained with 10 μM DCFH-DA dye (Beyotime, China) and incubated at 37 °C in the dark for 30 min. After washing with PBS, single-cell suspensions were collected and ROS levels were assessed using a flow cytometer (excitation wavelength, 488 nm; emission wavelength, 525 nm). Cell apoptosis analysis: Cells were stained using an Annexin V-FITC/PI dual staining kit (BD Biosciences, USA), and apoptosis was analyzed by detecting FITC and PI fluorescence signals with a flow cytometer. The apoptosis rate was then analyzed using FlowJo software. PD-L1 and CRT protein expression detection: After 3 PBS washes, cells were stained with anti-PD-L1 antibody (1:200, Proteintech, FITC-65081) and anti-CRT antibody (1:200, Proteintech, 27298-1-AP). In addition, isolated and treated splenocytes were stained with phycoerythrin-conjugated anti-mouse NK1.1 antibody (BioLegend), FITC-conjugated anti-CD3 antibody, and allophycocyanin-conjugated anti-CD8 antibody (BioLegend). After 30 min of incubation at room temperature in the dark, cells were washed 3 times with PBS. PD-L1 and CRT expression levels were analyzed using a flow cytometer, and the data were further analyzed with FlowJo software.

### LDH release assay

For natural killer (NK) cell killing activity detection, splenocytes were cocultured with Mec25 esophageal cancer cells at effector-to-target ratios (E:T) of 100:1, 50:1, and 25:1 for 4 h. Cell cytotoxicity was measured using a lactate dehydrogenase (LDH) cytotoxicity assay kit (Beyotime, Shanghai), and the cytotoxicity was calculated using the formula: Cytotoxicity (%) = [(experimental release OD − spontaneous release OD)/(maximum release OD − spontaneous release OD)] × 100%. In the CTL killing activity assay, splenocytes were incubated with Mec25 cell lysates for 48 h and then cocultured with Mec25 cells at an E:T ratio of 100:20 for 4 h. LDH cytotoxicity was measured as described above.

### Statistical analysis

All experimental data were analyzed using SPSS 19.0 and GraphPad Prism 8.0. Results are presented as mean ± standard error of the mean (SEM). Group comparisons used Student’s *t* test, with statistical significance set at *P* < 0.05, *P* < 0.01, and *P* < 0.001. All experiments were repeated at least 3 times to ensure accuracy and reproducibility.

## Results

### Preparation and characterization of US NBs

**Fig. 2.**
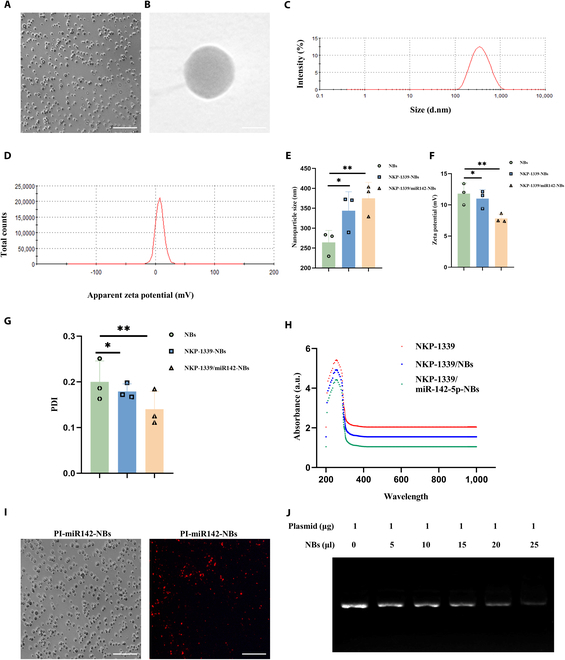
Preparation and characterization of NKP-1339/miR-142 NBs. (A) Morphology of NKP-1339/miR-142-NBs observed by optical microscopy (scale bar = 10 μm). (B) Morphological features of NBs were observed using transmission electron microscopy (80,000× magnification, scale bar = 200 nm). (C) Particle size distribution of NKP-1339/miR-142-NBs. (D) Zeta potential analysis of the surface charge of the NBs. (E) Particle size analysis of various modified nanobubble groups. (F) Zeta potential analysis of various modified nanobubble groups. (G) Polydispersity index (PDI) analysis of various modified nanobubble groups. (H) UV–Vis absorption spectra of NKP-1339/miR-142-NBs and free NKP-1339 showing no significant changes. (I) Red fluorescence of propidium iodide-stained miR-142 plasmid (PI-miR-142) on the surface of miR-142-NBs was observed using fluorescence microscopy (magnification 200×, scale bar = 10 μm). (J) Agarose gel electrophoresis confirming the loading capacity of the NBs for miR-142 plasmid.

NKP-1339/miR-142-NBs were synthesized by thin-film hydration and mechanical oscillation. Transmission electron microscopy revealed that the NBs were spherical with a uniform size distribution and smooth outer membranes (Fig. 2A and B). The particle size and zeta potential of NKP-1339/miR-142-NBs are shown in Fig. 2C and D. The results showed that the average particle sizes of NBs, NKP-1339-NBs, and NKP-1339/miR-142-NBs were 264.23 ± 17.39, 343.93 ± 27.32, and 374.87 ± 23.23 nm, respectively (Fig. 2E). The average zeta potentials were 11.8 ± 0.99, 11.0 ± 0.819, and 7.82 ± 0.406 mV (Fig. 2F), and the polydispersity indices (PDIs) were 0.200 ± 0.026, 0.179 ± 0.0096, and 0.140 ± 0.022, indicating good dispersion and stability (Fig. 2G). The UV–Vis absorption spectrum of NKP-1339/miR-142-NBs closely resembled that of free NKP-1339, suggesting that the NKP-1339 chromophore remained unchanged upon encapsulation (Fig. 2H). NBs loaded with PI-miR-142 plasmid exhibited red fluorescence on their surface (Fig. 2I). Using the membrane dialysis method, the encapsulation efficiency of NKP-1339 in NKP-1339/miR-142-NBs was determined to be 45.38% ± 3.01%. As shown in the gel electrophoresis results, no visible band appeared when the nanobubble (NB) volume reached 25 μl, indicating that 1 μg of miR-142 plasmid represented the maximum DNA loading capacity for 25 μl of NBs (Fig. 2J). These findings confirmed the morphology, particle size, surface charge, and plasmid loading efficiency of NKP-1339/miR-142-NBs, demonstrating their stability and loading capacity at the nanoscale. Therefore, NBs may serve as an effective delivery system for NKP-1339 and miR-142-5p.

### Pharmacokinetics and tissue distribution of US NBs: Prolonged circulation time and increased accumulation in tumor

**Fig. 3.**
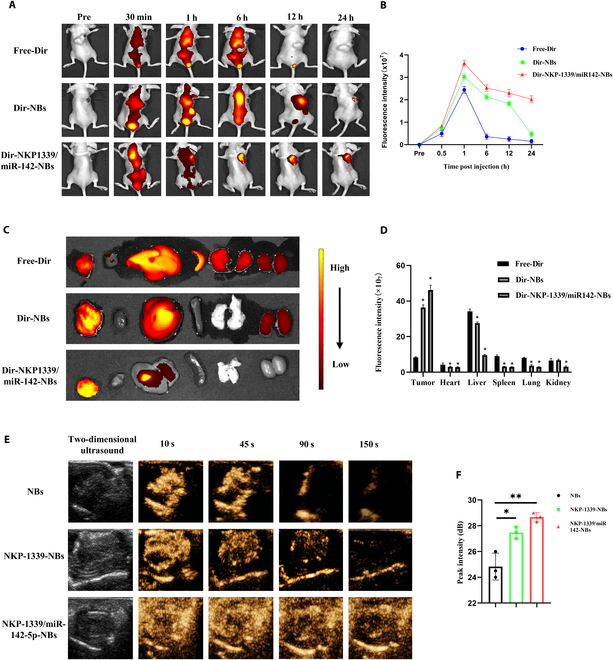
Pharmacokinetic analysis and tissue distribution of NKP-1339/miR-142-NBs+US. (A) Fluorescence distribution in tumor-bearing mice of different groups (Free-Dir, Dir-NBs, and Dir-NKP-1339/miR-142-NBs+US) was detected by the in vivo imaging system (IVIS). (B) Quantitative analysis of tissue fluorescence intensity from panel (A). (C) Fluorescence imaging of organs at 24 h postinjection for each group. (D) Quantitative analysis of tumor and organ fluorescence intensity from panel (C). (E) Contrast-enhanced US imaging results showing that NKP-1339/miR-142-NBs+US enhanced imaging and in vivo targeted detection. (F) Quantitative analysis of peak intensity in tumor regions for different groups. *P < 0.05, **P < 0.01.

To investigate the pharmacokinetic characteristics and tissue distribution of NKP-1339/miR-142-NBs combined with US treatment in tumor-bearing mice, the fluorescence distribution in different groups (Free-Dir, Dir-NBs, and Dir-NKP-1339/miR-142-NBs) was monitored in real-time using a small-animal imaging system. This was performed to verify whether the NBs could extend the circulation time of the drug and enhance its tumor-targeting accumulation. In vivo imaging showed a gradual increase in the fluorescence signal in tumor tissues, especially in the Dir-NKP-1339/miR-142-NBs group, which exhibited a considerably higher fluorescence intensity than the other groups. Six hours after injection, the fluorescence signal in the Free-Dir group notably decreased, while signals in the Dir-NBs and Dir-NKP-1339/miR-142-NBs groups persisted, with the latter group maintaining a high fluorescence level at the tumor site even after 24 h (Fig. 3A and B), indicating that the NBs markedly enhanced drug accumulation in the tumor, with the Dir-NKP-1339/miR-142-NBs combination showing the best targeting effect.

Organ imaging results revealed that fluorescence in the Free-Dir group was primarily concentrated in the tumor, followed by the liver and kidneys, suggesting that the drug might be cleared via hepatic and renal metabolism (Fig. 3C). In contrast, fluorescence intensity in the tumor tissue of the Dir-NBs and Dir-NKP-1339/miR-142-NBs groups was appreciably higher, being 4.36 and 5.54 times that of the free-Dir group, respectively (Fig. 3D), further confirming that US NBs can enhance drug targeting and tumor accumulation. To observe the retention and imaging properties of the NBs in vivo, CEUS was performed to monitor the distribution of the contrast agent within the tumor tissue. The results indicated that all 3 types of NBs exhibited good imaging capabilities (Fig. 3E). At 150 s postinjection, the contrast agent in the NBs group almost disappeared from the tumor, whereas in the NKP-1339-NBs and NKP-1339/miR-142-NBs groups, the contrast agent signals remained with fluorescence intensities 1.63 and 1.71 times that of the N-NB group, respectively (Fig. 3E and F). This suggests that US-targeted NBs can specifically accumulate at the tumor site and effectively prolong the drug retention time, further enhancing drug targeting and delivery efficiency.

The pharmacokinetic and tissue distribution results of this study demonstrate that NKP-1339/miR-142-NBs not only extend the drug’s circulation time but also markedly increase its tumor-targeting accumulation, while reducing nonspecific distribution in healthy tissues. This targeted enhancement and prolonged retention mechanism further validates the potential of NBs in tumor therapy.

### Antitumor effects of NKP-1339/miR-142-NBs combined with US

#### Tumor-suppressive effect of NKP-1339/miR-142-NBs

**Fig. 4.**
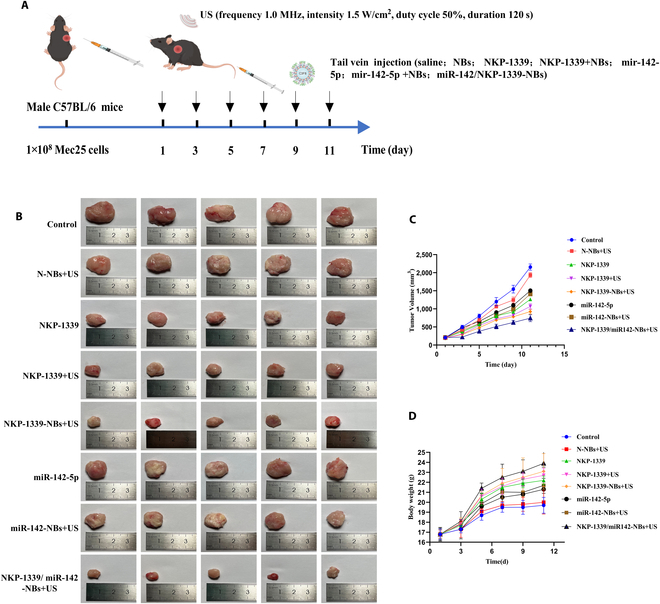
In vivo experiment on the anticancer effect of NKP-1339/miR-142-NBs combined with US treatment in esophageal cancer subcutaneous xenografts of mice. (*n* = 6 per group). (A) Schematic diagram of the in vivo treatment protocol for esophageal cancer-bearing mice. (B) Comparison of tumor sizes in mice from each group. (C) Tumor growth curves for mice in each group. (D) Changes in body weight of mice in each group.

The antitumor effects of NKP-1339/miR-142-NBs combined with US therapy were evaluated in an esophageal cancer tumor-bearing mouse model (*n* = 6 per group). The treatment protocol is outlined in Fig. 4A, and tumor growth and growth curves are presented in Fig. 4B and C. The experimental results demonstrated that the control group exhibited rapid tumor growth, whereas all treatment groups showed remarkably inhibited tumor volume and mass, with the NKP-1339/miR-142-NBs+US group showing the most pronounced antitumor effect.

In addition, the tumor volume increased over time, with the saline control group showing the fastest growth. Tumor inhibition rates revealed that the NKP-1339 and NKP-1339+US groups had remarkably higher tumor volume inhibition rates (*P* < 0.01). Notably, the NKP-1339-NBs+US group achieved a volume inhibition rate of 50.65% ± 4.54%, considerably outperforming the drug-only and US-only groups (*P* < 0.01), while the NKP-1339/miR-142-NBs+US group displayed the highest volume inhibition rate of 60.37% ± 6.77% (*P* < 0.01). Tumor weight analysis also indicated a reduction in tumor mass in all treatment groups compared with that in the control group. The NKP-1339-NBs+US and NKP-1339/miR-142-NBs+US groups showed the most significant tumor mass inhibition rates of 67.65% ± 1.36% and 79.72% ± 0.1%, respectively (Table). Furthermore, the mouse body weight data showed that the control and N-NBs+US groups showed a gradual decrease in weight gain, whereas the other treatment groups exhibited an increased weight gain rate (Fig. 4D). In conclusion, the NKP-1339/miR-142-NBs combined with US treatment demonstrated a synergistic antitumor effect, with the NKP-1339/miR-142-NBs+US group showing the most significant therapeutic effect and offering a potential new strategy for esophageal cancer treatment.

**Table.** The tumor inhibition rate of tumor volume and weight in each group

Groups	Tumor volume (mm^3^)	Tumor weight (g)	Volume inhibition rate (%)	Weight inhibition rate (%)
Control	1,876.67 ± 24.94	3.53 ± 0.13	0	0
N-NBs+US	1,703.67 ± 56.45	2.68 ± 0.16	7.42 ± 2.77*	18.79 ± 1.29*
NKP-1339	1,366.67 ± 96.09	1.79 ± 0.11	32.39 ± 2.08**	49.37 ± 1.19**
NKP-1339-NBs	1,185 ± 139.37	1.54 ± 0.08	43.07 ± 4.84**	56.52 ± 1.25**
NKP-1339-NBs+US	926.67 ± 113.8	1.14 ± 0.08	50.65 ± 4.54**	67.65 ± 1.36**
miR-142-5p	1,647.33 ± 145.05	2.14 ± 0.06	19.81 ± 1.12**	39.42 ± 1.14**
miR-142-NBs+US	1,447.67 ± 55.19	1.93 ± 0.04	24.63 ± 2.3**	45.26 ± 0.89**
miR-142/NKP-1339-NBs+US	745 ± 167.1	0.71 ± 0.03	60.37 ± 6.77**	79.72 ± 0.1**

* *P* < 0.05 vs. control group.

** *P* < 0.01 vs. control group.

#### NKP-1339/miR-142-NBs+US induces apoptosis in esophageal cancer cells

To evaluate the apoptotic effects of NKP-1339/miR-142-NBs combined with US treatment on tumor cells, H&E staining was performed to assess pathological changes in tumor tissues, and the TUNEL assay was employed to measure apoptosis levels in tumor cells. H&E staining showed varying degrees of cellular degeneration and necrosis across all treatment groups, with the NKP-1339/miR-142-NBs+US group exhibiting the most pronounced pathological changes (Fig. S1A). In the control group, the tumor cells were highly proliferative, characterized by enlarged and intensely stained nuclei, a considerably increased nuclear-to-cytoplasmic ratio, and notable cellular atypia. Compared with the control group, the NKP-1339 group showed a significant increase in the proportion of apoptotic cells. Furthermore, the apoptosis levels in the NKP-1339-NBs+US group were higher than those in the NKP-1339+US and N-NBs+US groups (*P* < 0.05, Fig. S1B and E). Notably, the NKP-1339/miR-142-NBs+US group demonstrated the highest levels of apoptosis, which were higher than those in the miR-142-5p, miR-142-NBs+US, and NKP-1339-NBs+US groups (*P* < 0.05). Flow cytometry results further confirmed that compared with the control group, the NKP-1339 group had a higher proportion of apoptotic cells. The apoptosis rate in the NKP-1339-NBs+US group was 12.82%, which was higher than those in the NKP-1339+US and N-NBs+US groups (*P* < 0.01). The NKP-1339/miR-142-NBs+US group had an apoptosis rate of 15.09%, surpassing that of the miR-142-NBs+US and NKP-1339-NBs+US groups (*P* < 0.05, Fig. S1D and H).

To further investigate the role of NKP-1339/miR-142-NBs+US combination therapy in the apoptotic mechanism, immunohistochemistry was performed to detect the expression of the apoptosis-related proteins Bax and Bcl-2. Bax and Bcl-2 proteins promote and inhibit programmed cell death, respectively, and play pivotal roles in regulating apoptosis. Compared with that in the control group, Bax protein expression was appreciably up-regulated in the NKP-1339 group, and Bax levels in the NKP-1339-NBs+US group were higher than those in the NKP-1339+US and N-NBs+US groups (Fig. S1C and F). Notably, the NKP-1339/miR-142-NBs+US group exhibited the highest Bax protein expression across all treatment groups (*P* < 0.05, Fig. S1F). In contrast, Bcl-2 protein expression decreased in all groups, showing a negative correlation with Bax expression (Fig. S1C and G). Moreover, Western blot (WB) and qPCR results further confirmed the expression patterns of Bax and Bcl-2, which were consistent with the immunohistochemical findings (Fig. S1I to M).

These results suggested that NKP-1339/miR-142-NBs combined with US therapy appreciably promoted tumor cell apoptosis. The underlying mechanism may involve the up-regulation of Bax expression and the down-regulation of Bcl-2 expression, which appears to be closely linked to the mitochondrial damage-induced apoptotic pathway.

### NKP-1339/miR-142-NBs+US induced antitumor immune responses

#### Verification of miR-142-5p targeting PD-L1

To explore the regulatory effect of miR-142-5p on PD-L1 protein expression, plasmids GV268, GV268-miR-142-5p, and synthetic oligonucleotides (miR mimic NC, miR-142-5p mimic, miR inhibitor NC, and miR-142-5p inhibitor) of equal concentrations were transfected into esophageal cancer cells. After 48 h, the cells were collected, and PD-L1 protein levels were detected using WB. The results showed that compared with that in the GV268-transfected group, PD-L1 protein expression was substantially reduced following miR-142-5p transfection. Similarly, compared with the miR mimic NC group, transfection with the miR-142-5p mimic led to the down-regulation of PD-L1 protein expression. However, transfection with the miR-142-5p inhibitor resulted in considerably higher PD-L1 expression than that in the miR inhibitor NC group (Fig. S2B and C). These findings suggest that miR-142-5p and its mimic suppress PD-L1 protein expression, whereas its inhibitor promotes PD-L1 expression. Further validation by qPCR confirmed that miR-142-5p substantially reduced PD-L1 mRNA levels, which was consistent with the trend in protein expression (Fig. S2D and E).

Flow cytometry confirmed that miR-142-5p considerably suppressed the surface expression of PD-L1, whereas its inhibitor promoted PD-L1 expression (Fig. S2F and G). In vivo experiments showed that both miR-142-5p-NBs+US and NKP-1339/miR-142-NBs+US treatments considerably reduced PD-L1 expression in tumor tissues (Fig. S2H), consistent with the WB and qPCR results (Fig. S2I to L). In summary, miR-142-5p down-regulated PD-L1 expression by targeting and inhibiting its expression, thereby potentially exerting a significant antitumor effect. This mechanism was validated by both in vitro and in vivo experiments.

#### NKP-1339/miR-142-NBs increased CTL responses

To further investigate the antitumor activity of immune checkpoint inhibitors combined with drugs, and to assess the antitumor immune activity of immune checkpoint blockade, flow cytometry was used to evaluate the proliferative capacity of splenic lymphocytes from mice. Splenic lymphocytes were labeled with a carboxyfluorescein diacetate succinimidyl ester fluorescent probe, which was evenly distributed among the progeny cells as they divided, with a gradual reduction in the fluorescence intensity. Subsequently, flow cytometry was performed to assess lymphocyte proliferation in each group.

**Fig. 5.**
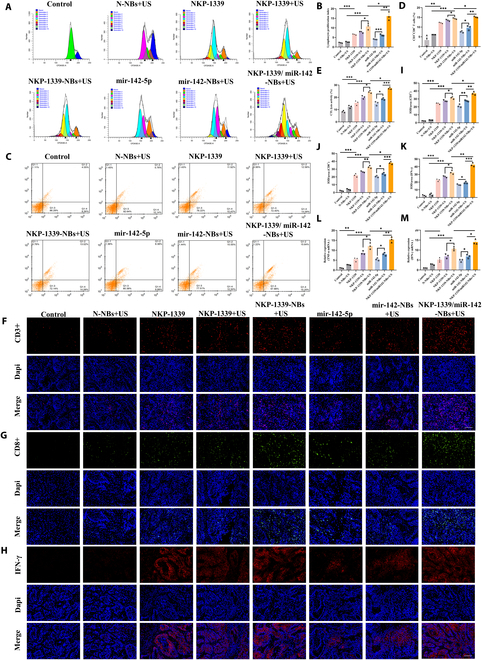
Mechanism on immune response of NK cell and macrophage induced by NKP-1339/miR-142-NBs combined with US treatment. (A and B) Flow cytometry analysis of the in vitro proliferation activity and proliferation index of splenic lymphocytes in each group. (C and D) Flow cytometry analysis of the proportion of CTL cells in each group and quantitative analysis. (E) Lactate dehydrogenase (LDH) release assay to evaluate the cytotoxic activity of CTL cells in vitro. (F to K) Immunofluorescence analysis of tumor tissue for the infiltration of CD3^+^ T CD8^+^ T cells and the expression of IFN-γ, along with semiquantitative analysis (200×, scale bar = 100 μm). (L and M) mRNA expression of TNF-α and IFN-γ in tumor tissues. *P < 0.05, **P < 0.01, ***P < 0.001.

Compared with that in the control group, the proliferative activity of lymphocytes in the NKP-1339 group showed an increasing trend, whereas the cell proliferation index in the NKP-1339-NBs+US group was higher than that in the NKP-1339+US and N-NBs+US groups (*P* < 0.05, Fig. 5A and B). Notably, the NKP-1339/miR-142-NBs+US group exhibited the highest in vitro proliferation index, which was approximately 7 times that of the control group and 1.5 times that of the NKP-1339-NBs+US group (*P* < 0.05), and was superior to those of the miR-142-NBs+US group and other single-treatment groups. These results suggest that NKP-1339/miR-142-NBs combination treatment can enhance the proliferative capacity of mouse lymphocytes.

Further analysis of the proportion of CD3^+^ CD8^+^ T cells revealed a trend consistent with the proliferation index. The proportion of CTLs in the NKP-1339-NBs+US group was higher than that in the N-NBs+US and NKP-1339+US groups (*P* < 0.05). The NKP-1339/miR-142-NBs+US group had the highest proportion of CD3^+^ CD8^+^ T cells (15.64 %) (*P* < 0.05, Fig. 5C and D). Further evaluation of the CTL killing ability through the LDH release assay showed that, compared with that of tumor cells cocultured with CTLs, the killing rate of target cells was higher in the NKP-1339-NBs+US group, whereas the NKP-1339/miR-142-NBs+US group reached the maximum value, which was consistent with the changes in the proportion of CD8^+^ T cells (Fig. 5E).

To verify the T cell-mediated immune response in the TME, immunofluorescence staining was used to detect the protein expression levels of CD3^+^ CD8^+^ lymphocytes and IFN-γ in tumor tissues. As shown in Fig. 5F to K, the expression of CD3^+^, CD8^+^, and IFN-γ in the NKP-1339 group was higher than in the control group (*P* < 0.001). Furthermore, the expression of CD3^+^, CD8^+^, and IFN-γ in the NKP-1339-NBs+US group was further enhanced compared with that in the N-NBs+US and NKP-1339+US groups (*P* < 0.05). The NKP-1339/miR-142-NBs+US group exhibited the highest levels of CD3^+^, CD8^+^, and IFN-γ expression, considerably higher than in all other groups (Fig. 5F to K). These results were consistent with the trends observed in the flow cytometry and LDH release assays.

Activation of cellular immunity can subsequently increase cytokine production, and the up-regulation of IFN-γ and TNF-α expression can trigger the maturation of DCs. Therefore, TNF-α and IFN-γ, which are crucial for immune cell activation, were investigated. RT-qPCR analysis revealed that mRNA expression levels of TNF-α and IFN-γ were consistent with their respective protein expression trends. The NKP-1339/miR-142-NBs+US group exhibited markedly higher mRNA levels of TNF-α and IFN-γ compared with the NKP-1339-NBs+US and other treatment groups (*P* < 0.05, Fig. 5L and M).

The above results indicate that NKP-1339/miR-142-NBs combined with US treatment can considerably promote CD8^+^ T cell proliferation and cytotoxicity, enhance the secretion of immune-related molecules such as TNF-α and IFN-γ, and further strengthen the tumor immune response. This antitumor immune activity may be achieved through the blockade of the programmed death-1 (PD-1)/PD-L1 pathway, enhancing the function of CTLs. These findings provide strong evidence for the application of targeted nanobubble-based drug delivery systems in tumor immunotherapy.

### NKP-1339/miR-142-NBs+US induced ICD mediated by mitochondrial dysfunction

#### NKP-1339/miR-142-NBs+US induced ICD

**Fig. 6.**
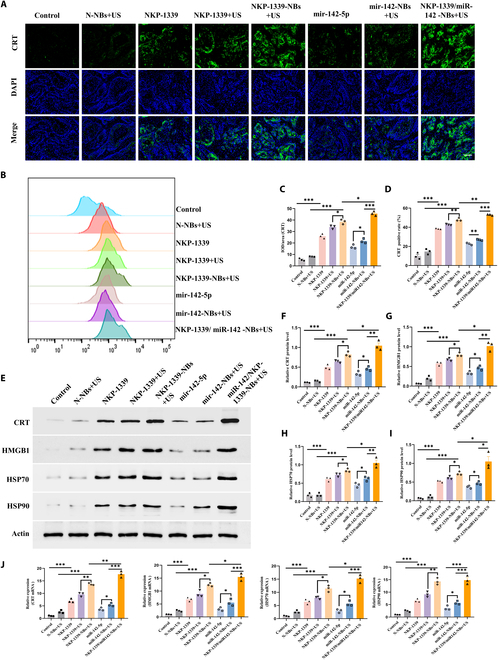
Mechanism on augmented ICD induced by NKP-1339/miR-142-NBs combined with US treatment. (A and C) Immunofluorescence analysis of CRT exposure on the surface of tumor cells in different treatment groups and semiquantitative analysis (200×, scale bar = 100 μm). (B and D) Flow cytometry analysis of CRT expression levels in each group and quantitative analysis. (E to I) Western blot analysis of CRT, HMGB1, HSP70, and HSP90 release levels and semiquantitative analysis. (J) qPCR analysis of mRNA expression levels of CRT, HMGB1, HSP70, and HSP90. *P < 0.05, **P < 0.01, ***P < 0.001.

CRT externalization is a key marker of ICD, and its exposure can trigger the phagocytosis of apoptotic tumor cells by macrophages in the TME, thereby activating an immune response. To explore the potential of NKP-1339/miR-142-NBs combined with US treatment to induce ICD, the expression and externalization of CRT were assessed using immunofluorescence, flow cytometry, and WB. As shown in Fig. 6A and C, all treatment groups (NKP-1339, NKP-1339+US, NKP-1339-NBs+US, and NKP-1339/miR-142-NBs+US) considerablypromoted the externalization of CRT onto tumor cell membranes, with the strongest green fluorescence signal observed in the NKP-1339/miR-142-NBs+US group, indicating the highest level of CRT externalization. Flow cytometric analysis revealed that CRT externalization in the NKP-1339/miR-142-NBs+US group reached its highest level of 53.39% (*P* < 0.01, Fig. 6B and D), which was markedly higher than that in the other groups. WB further confirmed that CRT protein expression was enhanced in the combined treatment group (*P* < 0.05, Fig. 6E and F).

HMGB1 release is another important feature of ICD that further enhances antitumor immunity by activating dendritic and T cells in the immune system. As shown in Fig. 6E, WB analysis indicated that, compared with the control group, NKP-1339 induced the expression of HMGB1. The HMGB1 expression level in the NKP-1339-NBs+US group was further elevated compared to that in the NKP-1339 and NKP-1339+US groups (*P* < 0.05, Fig. 6E and G). Notably, the NKP-1339/miR-142-NBs+US group exhibited the highest expression of HMGB1, considerably higher than that in the miR-142-5p, miR-142-NBs+US, and NKP-1339-NBs+US groups (*P* < 0.05), indicating that the combined treatment markedly enhanced the ICD response.

HSP70 and HSP90 are critical indicators of cellular stress responses and play key roles in ICD. The externalization of HSP70 on the cell surface enhances immune cell recognition and activates antitumor immune responses, while the release of HSP90 participates in regulating the DAMPs’ signaling pathway. WB results showed that compared with that in the control group, HSP70 expression was increased in the NKP-1339 group and was further enhanced in the NKP-1339+US group (*P* < 0.05). The expression of HSP70 was even higher in the NKP-1339-NBs+US group (Fig. 6E and H). Most importantly, HSP70 expression peaked in the NKP-1339/miR-142-NBs+US group and was higher than that in the miR-142-5p, miR-142-NBs+US, and NKP-1339-NBs+US groups (*P* < 0.05, Fig. 6H), further confirming the immunogenicity of the combined treatment. The expression pattern of HSP90 was consistent with that of HSP70 (Fig. 6I). The qPCR results for CRT, HMGB1, HSP70, and HSP90 showed trends similar to those observed using WB (Fig. 6J), further validating the ICD effect of the combined treatment.

These results indicate that NKP-1339/miR-142-NBs combined with US treatment can effectively induce ICD in tumor cells, mainly through CRT externalization, HMGB1 release, and the up-regulation of HSP70 and HSP90, thereby further activating antitumor immune responses. The combined treatment group showed significant improvements in all ICD markers.

#### NKP-1339/miR-142-NBs+US enhanced DC cell maturation

An increasing body of research suggests that the activation of ICD facilitates the exposure of tumor-associated antigens and stimulates DC activation, leading to tumor-specific T cell infiltration through a dramatic surge in DAMPs. To evaluate T cell-mediated immune responses, immunofluorescence staining was used to assess the infiltration of CD80^+^ CD86^+^ cells into tumor tissues. As depicted in Fig. S3A and B, and D and E, the NKP-1339 group exhibited a higher number of CD80^+^ CD86^+^ cells than the control group (*P* < 0.001, Fig. S3D and E). Additionally, the NKP-1339-NBs+US group demonstrated notably enhanced expression of CD80^+^ CD86^+^ proteins compared with the N-NBs+US and NKP-1339+US groups (*P* < 0.05, Fig. S3D and E). The NKP-1339/miR-142-NBs+US group showed the highest levels of CD80^+^CD86^+^ expression, which were higher than those observed in the other treatment groups (*P* < 0.05, Fig. S3D and E), indicating that the combined treatment more effectively promoted DC maturation.

NK cell activation plays a pivotal role in the antitumor immune response induced by ICD. Flow cytometric analysis of mouse spleen cells revealed a higher proportion of NK cells in the NKP-1339+US group than in the control group, with the NKP-1339-NBs+US group showing a further increase in the NK cell proportion (*P* < 0.05). The NKP-1339/miR-142-NBs+US group showed the highest proportion of NK cells (*P* < 0.05, Fig. S3C and F). Additionally, NK cell cytotoxicity against tumor cells, as assessed using an LDH release assay, followed the same trend as NK cell proportions, with the NKP-1339/miR-142-NBs+US group exhibiting the most significant cytotoxicity (*P* < 0.05, Fig. S3G).

#### NKP-1339/miR-142-NBs+US induced ICD mediated by mitochondrial structural and functional disruptions

##### NKP-1339/miR-142-NBs+US induced mitochondrial oxidative stress and dysfunction

**Fig. 7.**
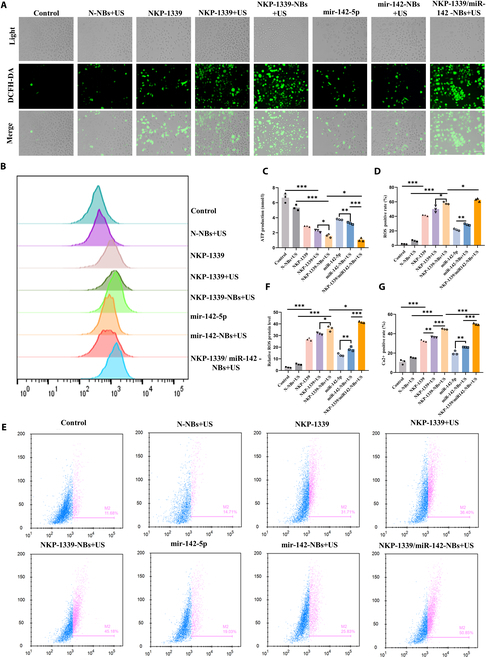
NKP-1339/miR-142-NBs combined with US treatment synergistically disrupt mitochondrial function via concurrent ATP depletion, ROS burst, and calcium dysregulation. (A and D) Immunofluorescence staining to detect ROS levels within esophageal cancer cells and semiquantitative analysis (200×, scale bar = 100μm). (B and F) Flow cytometry-based quantification of ROS-positive cell percentage. (C) Mitochondrial ATP levels measurement. (E to G) Flow cytometry analysis of calcium ion concentration changes and quantitative analysis. *P < 0.05, **P < 0.01, ***P < 0.001.

ATP production is a central function of mitochondria and serves as the basis for all cellular physiological activities. ATP generation relies on the mitochondrial electron transport chain, and its efficiency not only directly affects the cellular energy supply but also closely correlates with the production of ROS and calcium homeostasis. When ATP production decreases, the efficiency of the electron transport chain decreases, leading to increased electron leakage, which subsequently elevates ROS levels and disrupts calcium homeostasis. In changes to mitochondrial ATP levels, normal conditions maintain high ATP production to meet energy demands, whereas under damage conditions, ATP generation declines and leakage increases. During immune activation, ATP leakage can serve as an immune signal, enhancing the antitumor effects. Analysis of mitochondrial ATP levels revealed that ATP generation in the control and N-NBs+US groups was relatively high. However, in the NKP-1339 group, ATP levels were considerably reduced, indicating mitochondrial damage, with further decreases observed in the NKP-1339+US group. The ATP content in the NKP-1339-NBs+US group was lower (*P* < 0.05, Fig. 7C), and the ATP levels in the NKP-1339/miR-142-NBs+US group were the lowest (*P* < 0.05), suggesting that the combined treatment exacerbates mitochondrial damage.

ROS and Ca^2+^ are key regulators of mitochondrial function, influencing mitochondrial status through oxidative stress and calcium homeostasis, and also play important roles in regulating mitochondrial fission and fusion [18]. Mitochondria are the primary sources of ROS, which are by-products of ATP production. When present in appropriate amounts, ROS participate in signaling, but excess ROS induces oxidative damage. Excessive ROS can lead to calcium overload, triggering mitochondrial permeability transition (MPT) and ROS release, creating a vicious cycle [19]. Mitochondrial damage is often accompanied by excessive ROS production, leading to mitochondrial dysfunction and cellular stress. Intracellular ROS levels were examined using immunofluorescence staining. The results showed that ROS generation in the NKP-1339 group was increased compared with that in the control group and was further elevated in the NKP-1339+US and NKP-1339-NBs+US groups. Notably, the NKP-1339/miR-142-NBs+US group exhibited the highest ROS fluorescence intensity, which was higher than that of all other groups (*P* < 0.05, Fig. 7A and D), indicating that the combination of the drug and gene-loaded NBs under US exposure considerably induced oxidative stress in tumor cells. Subsequently, flow cytometry was used to quantify the cellular ROS levels, and the results were consistent with the immunofluorescence trends. Compared to the control and empty NBs groups, the NKP-1339 group showed a significant increase in the proportion of ROS-positive cells, with the NKP-1339-NBs+US group showing further elevation compared to the N-NBs+US and NKP-1339+US groups (*P* < 0.05). The highest proportion of ROS-positive cells (60.17 %) was observed in the NKP-1339/miR-142-NBs+US group, indicating a strong oxidative stress response (*P* < 0.05, Fig. 7B and F).

Mitochondria maintain homeostasis by regulating calcium ion flux, and calcium signaling plays a critical role in the regulation of ATP and ROS production. ROS enhances the activity of calcium channels, thereby establishing a positive feedback loop between ROS and calcium ions [20]. Calcium overload can lead to ROS accumulation and trigger MPT, halting ATP production [21]. This process further promotes mitochondrial division through the interaction between Drp1 and Fis1, exacerbating mitochondrial dysfunction and activating cell death signaling [22]. To investigate the calcium ion release under different treatment conditions, we analyzed the changes in calcium concentration in esophageal cancer cells using flow cytometry (Fig. 7E). The results demonstrated that calcium levels in the control group were maintained at low levels. In contrast, the NKP-1339 and NKP-1339+US groups exhibited a significant increase in the percentage of calcium-positive cells, suggesting that US facilitated mitochondrial calcium release and disrupted mitochondrial calcium homeostasis. In the combination treatment groups, the NKP-1339-NBs+US group showed a further increase in mitochondrial calcium levels compared to the NKP-1339+US group (*P* < 0.001, Fig. 7E and G), indicating that the synergistic effect of NBs and US exacerbated calcium release. The most significant change occurred in the NKP-1339/miR-142-NBs+US group, which had the highest percentage of calcium-positive cells (*P* < 0.001, Fig. 7G). These findings suggest that drug and gene combination therapy under US exposure may promote the opening of mitochondrial permeability transition pores (mPTPs), leading to a massive release of mitochondrial calcium stores into the cytoplasm, thus accelerating mitochondrial dysfunction.

This study demonstrates that the NKP-1339/miR-142-NBs combined with US treatment induced a decrease in mitochondrial ATP levels, markedly increased mitochondrial ROS levels, and promoted opening of the mPTP. The massive release of mitochondrial calcium stores into the cytoplasm disrupts calcium homeostasis, thereby forming a vicious cycle that accelerates mitochondrial damage. These results suggest that NKP-1339/miR-142-NBs-mediated mitochondrial dysfunction is closely associated with ROS generation and mPTP opening.

##### NKP-1339/miR-142-NBs+US induced mitochondrial fission disorder

Mitochondrial fission and fusion maintain morphology, function, and cellular homeostasis through dynamic balance. Fission isolates damaged mitochondria, while fusion optimizes the network to support ATP production and ROS clearance. These processes are regulated by ROS [23], calcium [24], and ATP [25] to ensure cellular homeostasis. Under normal conditions, DRP1 and FIS1 are key regulatory proteins for mitochondrial fission; DRP1 executes the fission process, and FIS1 acts as its outer membrane receptor. OPA1 and MFN2 regulate the fusion of inner and outer mitochondrial membranes, respectively, while maintaining mitochondrial network integrity. PGC1-α is a key regulator of mitochondrial biogenesis, promoting mitochondrial genesis, mitochondrial DNA (mtDNA) transcription, and replication by activating downstream proteins such as NRF1 and TFAM. The up-regulation of DRP1 and FIS1 typically indicates enhanced fission, whereas the down-regulation of OPA1 and MFN2 indicates impaired fusion. The activity of PGC1-α, NRF1, and TFAM is closely related to mitochondrial biogenesis and functional recovery, collectively regulating mitochondrial morphology, function, and homeostasis. In states of mitochondrial damage, fission is enhanced while fusion is suppressed, leading to mitochondrial fragmentation, increased ROS production, mtDNA release, and dysfunction. Mitochondrial dynamics are closely linked to immune activation, with activated immune cells exhibiting high fission rates. Mitochondrial fission has been shown to enhance the activation of the NLRP3 inflammasome and release pro-inflammatory cytokines [26], whereas the leakage of mtDNA can activate immune responses via the cyclic GMP-AMP synthase–stimulator of interferon genes pathway [27].

**Fig. 8.**
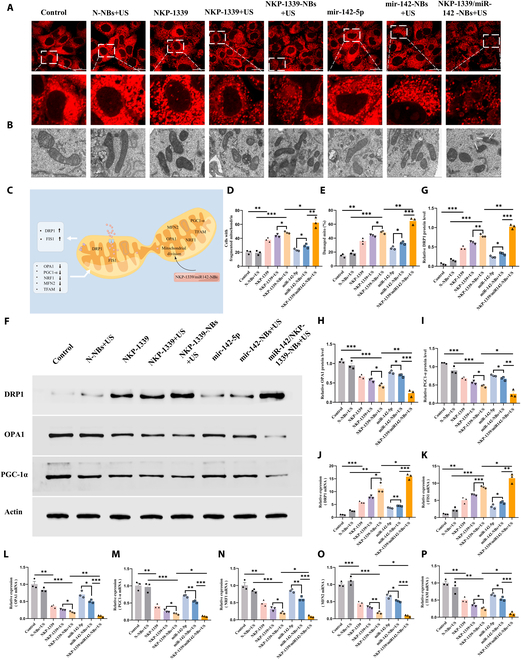
NKP-1339/miR-142-NBs combined with US treatment provoke pathological mitochondrial fission leading to structural disintegration in esophageal cancer cells. (A and D) Confocal fluorescence microscopy images of esophageal cancer cells showing mitochondrial fluorescence, with quantification of fragmented cells (scale bar = 50 μm). (C) Diagram of mitochondrial fission-regulating genes. (B and E) Representative TEM images (×20,000) of mitochondria in esophageal tumor tissues from different groups, and quantification of damaged mitochondria. Scale bar = 500 nm. (F to I) WB analysis of mitochondrial fission and fusion-related proteins, with semiquantitative analysis. (J to P) qPCR analysis of mRNA expression levels of mitochondrial function-related genes (DRP1, FIS1, OPA1, PGC1-α, NRF1, MFN2, and TFAM). *P < 0.05, **P < 0.01, ***P < 0.001.

To further “visualize” mitochondrial damage, the mitochondria-specific fluorescence probe MitoTracker was used to observe morphological changes under different treatment conditions. Fluorescence confocal microscopy showed that in the control and N-NBs+US groups, mitochondria exhibited typical linear and network structures with intact, continuous morphology, clear cristae, and minimal fission. In the NKP-1339 group, mitochondria showed mild fragmentation with localized breakage and a shrinking morphology. With the addition of US-assisted treatment (NKP-1339+US group), mitochondrial damage worsened, and some mitochondrial structures showed fragmentation. In the NKP-1339-NBs+US group, the mitochondria shifted considerably to a fragmented state, displaying more broken and dot-like structures. The most severe mitochondrial damage was observed in the NKP-1339/miR-142-NBs+US group, where mitochondria showed extensive fragmentation, lost the network structure entirely, and exhibited only dot-like and broken shapes (Fig. 8A and D).

To further assess mitochondrial ultrastructure, transmission electron microscopy was conducted on each group. In the Control and N-NBs+US groups, mitochondria maintained an intact architecture, characterized by well-preserved cristae and continuous double membranes. In contrast, the NKP-1339 group exhibited mild mitochondrial swelling and partial cristae disorganization. More pronounced structural damage was observed in the NKP-1339+US and NKP-1339-NBs+US groups, including disrupted membranes and widespread cristae loss. Notably, the NKP-1339/miR-142-NBs+US group displayed extensive mitochondrial injury, as evidenced by pronounced swelling, collapsed cristae, and rupture of the outer membrane—hallmarks indicative of severe mitochondrial destruction (Fig. 8B and E). Quantitative analysis further demonstrated a markedly increased proportion of damaged mitochondria in the codelivery+US group compared to all other groups (Fig. 8B and E).

WB analysis revealed that the mitochondrial fission-related protein DRP1 was markedly up-regulated in the NKP-1339 group, while the expression of fusion and biogenesis-related proteins OPA1 and PGC1-α was down-regulated. This trend was further exacerbated in the NKP-1339+US group, with DRP1 showing a more significant up-regulation (*P* < 0.01), and OPA1 and PGC1-α showing significant down-regulation (*P* < 0.05). In the NKP-1339-NBs+US group, DRP1 expression was the highest, and OPA1 and PGC1-α expression were the lowest (*P* < 0.05) (Fig. 8F to I).

Further qPCR analysis of mitochondrial function-related genes, including DRP1, FIS1, OPA1, PGC1-α, NRF1, MFN2, and TFAM, revealed that DRP1 and FIS1 were considerably up-regulated in all treatment groups (*P* < 0.05), while OPA1, PGC1-α, NRF1, MFN2, and TFAM levels gradually decreased, with the most significant changes observed in the NKP-1339/miR-142-NBs+US group compared to the miR-142-5p, miR-142-NBs+US, and NKP-1339-NBs+US groups (*P* < 0.05) (Fig. 8J to P). These results were consistent with the WB data, indicating that the combination treatment notably exacerbated mitochondrial dysfunction by promoting mitochondrial fission and inhibiting fusion and biogenesis.

In conclusion, the NKP-1339/miR-142-NBs combined with US treatment induced significant mitochondrial structural damage, up-regulated fission proteins (DRP1 and FIS1), and down-regulated fusion and biogenesis-related genes (OPA1, PGC1-α, NRF1, MFN2, and TFAM), suggesting that mitochondrial dysfunction may be linked to tumor cell apoptosis and ICD responses.

##### Mitochondrial fission disruption mediates ICD

**Fig. 9.**
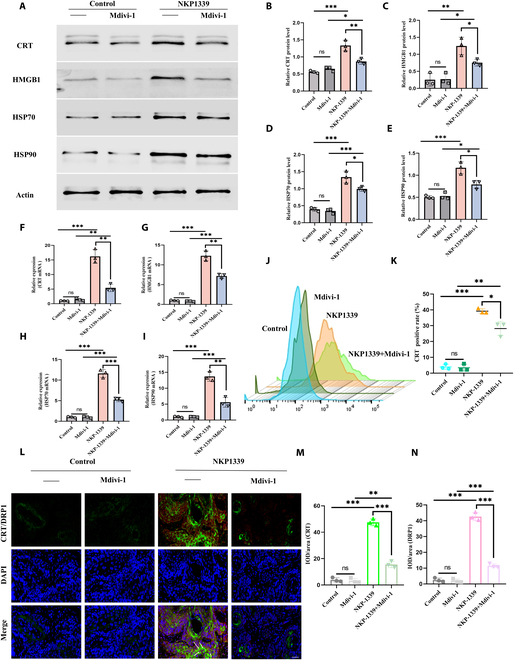
From mitochondrial fission suppression to enhanced ICD: The regulatory axis of NKP-1339 and fission inhibitors in tumor immunogenicity. (A to E) WB analysis of CRT, HMGB1, HSP70, and HSP90 protein expression levels, along with semiquantitative analysis. (F to I) qPCR analysis of CRT, HMGB1, HSP70, and HSP90 mRNA expression levels. (J and K) Flow cytometry analysis of CRT expression levels and quantitative analysis. (L to N) Representative immunofluorescence images showing Drp1 (red), calreticulin (CRT, green), and nuclei (blue) in different treatment groups. White arrows indicate representative tumor cells with synchronous activation , along with semiquantitative analysis. Scale bar = 20 μm. *P < 0.05, **P < 0.01, ***P < 0.001.

To further investigate the role of mitochondrial fission in NKP-1339-induced ICD, tumor cells were treated with a mitochondrial fission inhibitor. The experimental groups included control, Mdivi-1 (mitochondrial fission inhibitor treatment), NKP-1339, and NKP-1339+Mdivi-1 groups. The expression of ICD-related markers, including CRT, HMGB1, HSP70, and HSP90, was measured using WB, qPCR, and flow cytometry (Fig. 9A to I). Both WB and qPCR results indicated no significant differences in the expression of CRT, HMGB1, HSP70, and HSP90 between the Mdivi-1-treated and control groups (*P* > 0.05), suggesting that the mitochondrial fission inhibitor alone had limited effects on ICD markers. The NKP-1339 treatment group showed notably up-regulated expression of CRT, HMGB1, HSP70, and HSP90 (*P* < 0.01), indicating that NKP-1339 effectively induced ICD. However, in the NKP-1339+Mdivi-1 combination treatment group, the expression of these ICD markers was considerably lower than that in the NKP-1339-only treatment group (*P* < 0.05), suggesting that the mitochondrial fission inhibitor partially reversed the ICD-inducing effect of NKP-1339. Flow cytometry analysis of CRT exposure further confirmed that CRT externalization levels did not differ between the Mdivi-1 and control groups (*P* > 0.05), whereas CRT exposure was increased in the NKP-1339-treated group (*P* < 0.001) and decreased in the NKP-1339+Mdivi-1 group (*P* < 0.05, Fig. 9J and K).

To further elucidate the spatial correlation between mitochondrial fission and ICD, dual immunofluorescence staining for Drp1 (red) and CRT (green) was performed on tumor tissue sections. In the Control and Mdivi-1 groups, CRT expression was restricted to intracellular compartments without detectable membrane localization. In contrast, NKP-1339 treatment markedly increased cytoplasmic Drp1 puncta, indicating activated mitochondrial fission, and simultaneously promoted CRT externalization, forming a distinct green fluorescent ring along the plasma membrane (Fig. 9L to N). Although no colocalization was observed due to their distinct subcellular localizations, the 2 signals were consistently elevated within the same cells. Notably, in the NKP-1339+Mdivi-1 group, both Drp1 clustering and CRT membrane exposure were substantially reduced, suggesting that mitochondrial fission plays a functional role in promoting CRT translocation and ICD initiation. These results underscore the critical involvement of Drp1 in mediating NKP-1339-induced ICD responses.

These results suggest that NKP-1339 induces ICD by up-regulating ICD markers and that the inclusion of the mitochondrial fission inhibitor partially attenuates this effect, indicating a close relationship between NKP-1339-induced ICD and mitochondrial fission.

### Safety assessment of NKP-1339/miR-142-NBs

To evaluate the in vivo safety of NKP-1339/miR-142-NBs, H&E staining was used to examine histopathological changes in the major organs (heart, liver, spleen, lungs, and kidney) of mice in each group. The experimental results indicated that the organ structures of mice in all groups remained intact, with no significant signs of inflammation, necrosis, or other pathological damage. Tissue morphology was normal (Fig. S4A). Further measurements of the liver and kidney indices showed no statistically significant differences between the treatment and control groups (Fig. S4B and C). These data suggest that NKP-1339/miR-142-NBs did not affect the liver or kidney function during treatment.

In conclusion, NKP-1339/miR-142-NBs exhibited good biocompatibility and safety in tumor-bearing mice, with no significant pathological changes or toxic side effects, demonstrating a high level of safety and promising therapeutic potential.

## Discussion

US NBs are novel drug-delivery platforms with significant advantages in cancer therapy owing to their targeting ability and multifunctionality. With a nanoscale diameter (<500 nm), NBs accumulate at tumor sites via EPR effects, penetrate abnormal vascular barriers, and promote drug enrichment in the TME [28]. The positively charged surface of NBs interacts with the negatively charged TME, enhancing the targeted delivery efficiency [29]. Compared to traditional viral vectors, NBs are an ideal choice for delivering genes, chemotherapeutic agents, and immunotherapy molecules because of their low immunogenicity, ease of preparation, and good safety profile. The cationic lipid shell of NBs improves the drug-loading capacity and stability of miRNAs, preventing their rapid degradation in vivo [30]. Upon US activation, NBs utilize cavitation and sonoporation effects to enhance cell membrane permeability [31], improve tumor cell uptake of NKP-1339 and miR-142-5p, enable spatiotemporally controlled drug release, and minimize off-target toxicity to healthy tissues. Our team discovered that NBs effectively delivered Ce6 and miR-195, leading to successful cervical cancer treatment. In this study, we developed a multifunctional nanolipobubble system loaded with NKP-1339 and miR-142 for efficient delivery and precise release via US targeting. Pharmacokinetic studies showed that NKP-1339/miR-142-NBs US prolongs drug circulation, enhances accumulation in tumor tissues, and reduces nonspecific distribution in healthy tissues. This system improved NKP-1339 stability and reduced its systemic toxicity, offering an effective strategy for precise anticancer therapy.

ICD has gained attention as a combination cancer therapy that complements traditional treatments and immunotherapies. Studies have shown that various ICD inducers, such as chemotherapeutic agents, photosensitizers, and radiation, activate antitumor immune responses by inducing cellular stress, and stimulating the release of DAMPs, which inhibit cancer progression [32]. Increasing evidence suggests that mitochondria play a critical role in immune modulation [33]. Because of the lack of histone protective packaging, mtDNA is particularly vulnerable to stress-induced damage. Mitochondrial dysfunction, such as ROS generation, ATP leakage, and cytochrome c release, is associated with ICD activation [34]. Induction of mitochondrial stress exposes immunogenic mitochondrial antigens and regulates antitumor immune responses [35]. Moreover, mitochondrial stress amplifies stress signals by coupling with the ER, thereby enhancing antitumor immunity. Mitochondria are ideal targets for ICD inducers and provide insights into mitochondrial stress during ICD induction. Mitochondria are central regulators of cellular energy and are linked to cancer cell survival. Both exogenous and endogenous stressors damage the mitochondria, leading to excessive ROS production and oxidative stress. Oxidative stress impairs mitochondrial function, reduces ATP generation, triggers apoptosis, and releases DAMPs, further promoting ICD. ER and mitochondria maintain calcium homeostasis. Disruption of calcium homeostasis, particularly mitochondrial calcium overload, induces mitochondrial dysfunction, cell death, and ICD [36]. Furthermore, ROS imbalance disrupts mitochondrial dynamics, altering the expression of genes related to fission and biogenesis (e.g., DRP1, OPA1, and PGC1-α), further compromising mitochondrial function [37]. NKP-1339, a novel ICD inducer, exerts antitumor effects by depleting mitochondrial ATP, promoting ROS generation, disrupting mitochondrial calcium homeostasis, triggering mitochondrial dysfunction, and amplifying ICD effects. Additionally, DAMPs released by NKP-1339 enhanced antigen-presenting cell activity and activated DC maturation, further amplifying the immune response. These findings highlight the unique advantages of NKP-1339 in inducing ICD and enhancing antitumor immunity, thus supporting the development of NKP-1339-based ICD therapies.

Immune checkpoint inhibitor (ICI) therapy, which restores T cell activity by blocking the PD-1/PD-L1 pathway, is an important strategy in cancer immunotherapy to induce antitumor immune responses. However, the efficacy of single-agent ICI therapy is limited by poor immunogenicity, particularly in “cold tumors” with minimal immune infiltration. Therefore, combining ICI therapy with other treatments is necessary to convert “cold tumors” into “hot tumors” and enhance immune responses. For example, combining ICI with chemotherapy [38], radiotherapy, or targeted therapy [39] can improve the effectiveness of immunotherapy by enhancing antigen presentation, activating T cells, and reducing the number of immunosuppressive cells. miR-142, a key tumor-suppressive miRNA, regulates cell proliferation, invasion, and apoptosis and enhances T cell activity by down-regulating PD-L1 expression, thereby improving ICI efficacy. Studies have indicated that miR-142-5p alleviates the immunosuppressive TME by inhibiting PD-L1 expression. PD-L1 not only is highly expressed in various tumor cells but also plays a crucial immunosuppressive role in certain normal immune cells, such as activated T cells, B cells, and DCs, where it contributes to maintaining peripheral immune tolerance. Previous studies have demonstrated that PD-L1-deficient mice exhibit heightened T cell reactivity and increased susceptibility to autoimmune diseases in preclinical models [40]. Therefore, in theory, systemic delivery of miR-142-5p may lead to unintended down-regulation of PD-L1 in normal immune cells, potentially disrupting immune homeostasis and triggering immune overactivation or autoimmunity-related toxicity. However, to date, there is no direct clinical or preclinical evidence linking systemic miR-142-5p therapy to the onset of autoimmune diseases. Moreover, several miRNAs, including miR-142-5p, miR-155, and miR-200c, have been reported to down-regulate PD-L1 expression and enhance antitumor immunity [41,42]. In our study, we employed an ultrasound-responsive NB-based delivery system, which offers excellent tumor-targeting capability and spatiotemporally controlled release. This strategy reduces the nonspecific distribution of miRNAs in normal tissues [43]. Pharmacokinetic and biodistribution analyses further demonstrated that NKP-1339/miR-142-5p-NBs exhibited prolonged tumor retention and high intratumoral accumulation, while showing minimal distribution in major immune organs such as the spleen. These findings suggest that the systemic exposure of miR-142-5p is limited when delivered via NBs, thereby minimizing the risk of off-target effects in normal immune cells. Additionally, histopathological evaluations of major organs and serum biochemical analyses of liver and kidney function revealed no significant pathological alterations or systemic toxicity.

The combined application of chemotherapy and gene therapy has become a major focus in cancer treatment, as both strategies complement each other through different mechanisms and offer novel therapeutic options for refractory cancers [44]. Gene therapy achieves precise treatment by modulating oncogenes or restoring tumor suppressor genes [45], whereas chemotherapy combined with immunotherapy uses chemotherapeutic agents to kill cancer cells and immunotherapy to enhance immune system function. Some chemotherapy drugs induce ICD, making cancer cells more susceptible to immune recognition, whereas immune therapy boosts the immune response, kills cancer cells, and enhances immunity [46]. The combination of chemotherapy, immunotherapy, and gene therapy may be more effective than monotherapy by providing targeted destruction, reducing side effects, and improving the quality of life, while overcoming drug resistance and promoting durable responses [44]. In this study, the combination of NKP-1339 and miR-142-5P represents a typical example of complementary gene therapy and immunotherapy for combating the high invasiveness and resistance of esophageal cancer. NKP-1339 promotes antigen presentation by inducing mitochondrial dysfunction and releasing immunogenic death signals, whereas miR-142-5P enhances T cell activity by down-regulating PD-L1 expression, resulting in T cell proliferation and antitumor cytotoxicity through MHC-TCR activation. This synergy between chemotherapy, immunotherapy, and gene therapy demonstrates strong mechanistic complementarity and enhances antitumor efficacy.

ICD can effectively trigger tumor antigen release and initiate antitumor immune responses; however, studies have shown that without sufficient induction of memory T cells, the resulting immune activation may be transient and inadequate to prevent tumor recurrence [47]. Similarly, miRNA-mediated PD-L1 inhibition can effectively relieve immunosuppression and enhance T cell activation and cytokine production in the short term [48], but such strategies alone may fail to sustain robust immune responses in the absence of long-lived memory T cells [49]. These findings suggest that ICD-based therapies alone are insufficient for long-term tumor eradication, and additional strategies that promote immune memory formation are required. Recently, the phase Ib study by the Spanish Sarcoma Group (GEIS) demonstrates that the combination of doxorubicin, dacarbazine, and nivolumab is a tolerable and potentially effective first-line therapy for advanced leiomyosarcoma, showing a 34.4% objective response rate and a median progression-free survival of 7.2 months. The combination leverages ICD induced by doxorubicin (HMGB1/ATP release) and synergizing with nivolumab’s PD-1 blockade to enhance T cell activation and overcome tumor immune evasion in advanced leiomyosarcoma, indicating that ICD–ICI synergy may enhance T cell activation and reduce immune escape [50]. From a translational perspective, although this strategy exhibits a favorable safety profile and strong immunostimulatory activity in animal models, several critical challenges must be addressed before clinical application. Scalable and reproducible manufacturing of nanobubbles with consistent drug loading efficiency, size distribution, and ultrasound responsiveness is essential for industrialization. Moreover, the potential off-target effects of miR-142-5p—especially its impact on PD-L1 expression in normal immune cells—must be rigorously evaluated to mitigate risks of immune dysregulation or autoimmune toxicity. Additionally, human tumors exhibit substantial immunological heterogeneity, and further validation in patient-derived organoids or humanized mouse models is needed. These considerations are critical for future preclinical and clinical development.

In conclusion, this study introduced a combination of NKP-1339 and miR-142-5P using US-targeted NBs for esophageal cancer therapy, showing significant antitumor effects. Compared with individual treatments, combined therapy considerably enhanced efficacy. This was achieved through US NBs, which enhance drug penetration and local concentration in tumor tissues via the cavitation effect. Additionally, this combination triggers mitochondrial damage and dysfunction, inducing ICD, which promotes tumor-associated antigen release and activates the immune response. At the molecular level, NKP-1339/miR-142-5P-NBs reduce mitochondrial ATP production, increase ROS levels, and disrupt calcium homeostasis, resulting in mitochondrial damage. This damage disrupts mitochondrial dynamics, up-regulates the expression of ICD-related molecules, such as CRT and HMGB1, activates ICD signaling pathways, and enhances antitumor immune responses. Combined therapy also enhances NK cell function. miR-142, by down-regulating PD-L1 expression and blocking the PD-1/PD-L1 pathway, alleviates tumor-induced immune suppression, promoting CD3^+^ CD8^+^ T cell proliferation and restoring T cell activity via increased interferon-γ expression. Thus, NKP-1339 and miR-142 NBs combined US therapy induced mitochondrial dysfunction and ICD, enhanced CTL activity, and synergistically triggered immune responses. This study uncovers a new mechanism for combining chemotherapy–immunotherapy with gene-targeted therapy, demonstrating promising biological safety and therapeutic potential for esophageal cancer treatment.

## Data Availability

The datasets generated and/or analyzed during the current study are available from the corresponding author upon reasonable request.
