# Supplementary material for: Ultrasound-Targeted Nanobubbles Codelivering NKP-1339 and miR-142-5p for Synergistic Mitochondrial Immunogenic Cell Death and PD-L1 Inhibition in Cancer Therapy
Source: Biomater Res. 2025 Aug 8;29:0232. doi: 10.34133/bmr.0232 (PMC12334082; doi:10.34133/bmr.0232)
Supplement: Supplementary 1 — Figs. S1 to S4 Table S1 [file bmr.0232.f1.docx]

*Supporting Information*

**Ultrasound-Targeted Nanobubbles Co-delivering NKP-1339 and miR-142-5p for Synergistic Mitochondrial ICD and PD-L1 Inhibition in Cancer Therapy**

Yafei Zhang^1^, Chaoqi Liu^2,3^, Shuai Jin^4^, Liangyun Xie^1^, Qianwen Xiao^1^, Jun Yao^1*^

^1^The First Affiliated Hospital, and College of Clinical Medicine of Henan University of Science and Technology, Luoyang, China,471003

^2^Hubei Key Laboratory of Tumor Microenvironment and Immunotherapy, China Three Gorges University, Yichang, Hubei, China, 443002

^3^Medical College of China Three Gorges University, Yichang, Hubei, China, 443002

^4^The First Clinical Medical College of Three Gorges University, Center People’s Hospital of Yichang, Yichang, Hubei, China, 443008

* Correspondence: Jun Yao, yaojun74@163.com

**This file includes:**

Supporting Table S1

Supporting Figures S1 to S4

**Table S1** **Primer Sequences**

| **Gene** | **Primer** | **Sequences** |
| --- | --- | --- |
| miR-142-5p | Sense | GGTGGGTGCATAAAGTAGAAAGC |
|  | Antisense | CAGTGCAGGGTCCGAGGT |
| U6 | Sense | CTCGCTTCGGCAGCACA |
|  | Antisense | AACGCTTCACGAATTTGCGT |
| PD-L1 | Sense | TGCTGCATAATCAGCTACGG |
|  | Antisense | CCACGGAAATTCTCTGGTTG |
| CRT | Sense | CGGAAGACTGGGATGAACG |
|  | Antisense | TCAGGGTCAGGGATGTGCT |
| HMGB1 | Sense | CATCCTGGCTTATCCATTGGTG |
|  | Antisense | TCTTCTCATAAGGCTGCTTGTCAT |
| HSP70 | Sense | TGAACTACAAGGGCGAGAGCC |
|  | Antisense | GCCGCTGAGATGCGTTGAAGT |
| HSP90 | Sense | AGGACCAGGTTGCTAACTCCG |
|  | Antisense | CACACAATACTCATCAATGGGCTC |
| TNF-α | Sense | TCAACCTCCTCTCTGCCGTC |
|  | Antisense | GAGCAATGACTCCAAAGTAGACCTG |
| IFN-γ | Sense | CCATCGGCTGACCTAGAGAA |
|  | Antisense | GATGCAGTGTGTAGCGTTCA |
| β-actin | Sense | GGAGATTACTGCCCTGGCTCCTA |
|  | Antisense | GACTCATCGTACTCCTGCTTGCTG |
| DRP1 | Sense | ATGCCAGCAAGTCCACAGAA |
|  | Antisense | TGTTCTCGGGCAGACAGTTT |
| FIS1 | Sense | CAAAGAGGAACAGCGGGACT |
|  | Antisense | ACAGCCCTCGCACATACTTT |
| NRF1 | Sense | AGAAACGGAAACGGCCTCAT |
|  | Antisense | CATCCAACGTGGCTCTGAGT |
| OPA1 | Sense | ACCTTGCCAGTTTAGCTCCC |
|  | Antisense | TTGGGACCTGCAGTGAAGAA |
| PCG1-α | Sense | GCAGTCGCAACATGCTCAAG |
|  | Antisense | GGGAACCCTTGGGGTCATTT |
| MFN2 | Sense | TGCACCGCCATATAGAGGAAG |
|  | Antisense | TCTGCAGTGAACTGGCAATG |
| TFAM | Sense | TCCACAGAACAGCTACCCAA |
|  | Antisense | CCACAGGGCTGCAATTTTCC |

Abbreviations:miR-142-5p:MicroRNA-142-5p;PD-L1:Programmed Death-Ligand 1;CRT:Calreticulin;HMGB1：High Mobility Group Box 1;HSP70:Heat Shock Protein 70;HSP90:Heat Shock Protein 90;TNF-α:Tumor Necrosis Factor-alpha;IFN-γ:Interferon-gamma;β-actin:Beta-actin;DRP1:Dynamin-Related Protein 1;FIS1:Mitochondrial Fission 1 Protein;NRF1:Nuclear Respiratory Factor 1;OPA1:Optic Atrophy 1;PGC1-α:Peroxisome Proliferator-Activated Receptor Gamma Coactivator 1-alpha;MFN2:Mitofusin 2;TFAM:Mitochondrial Transcription Factor A

**
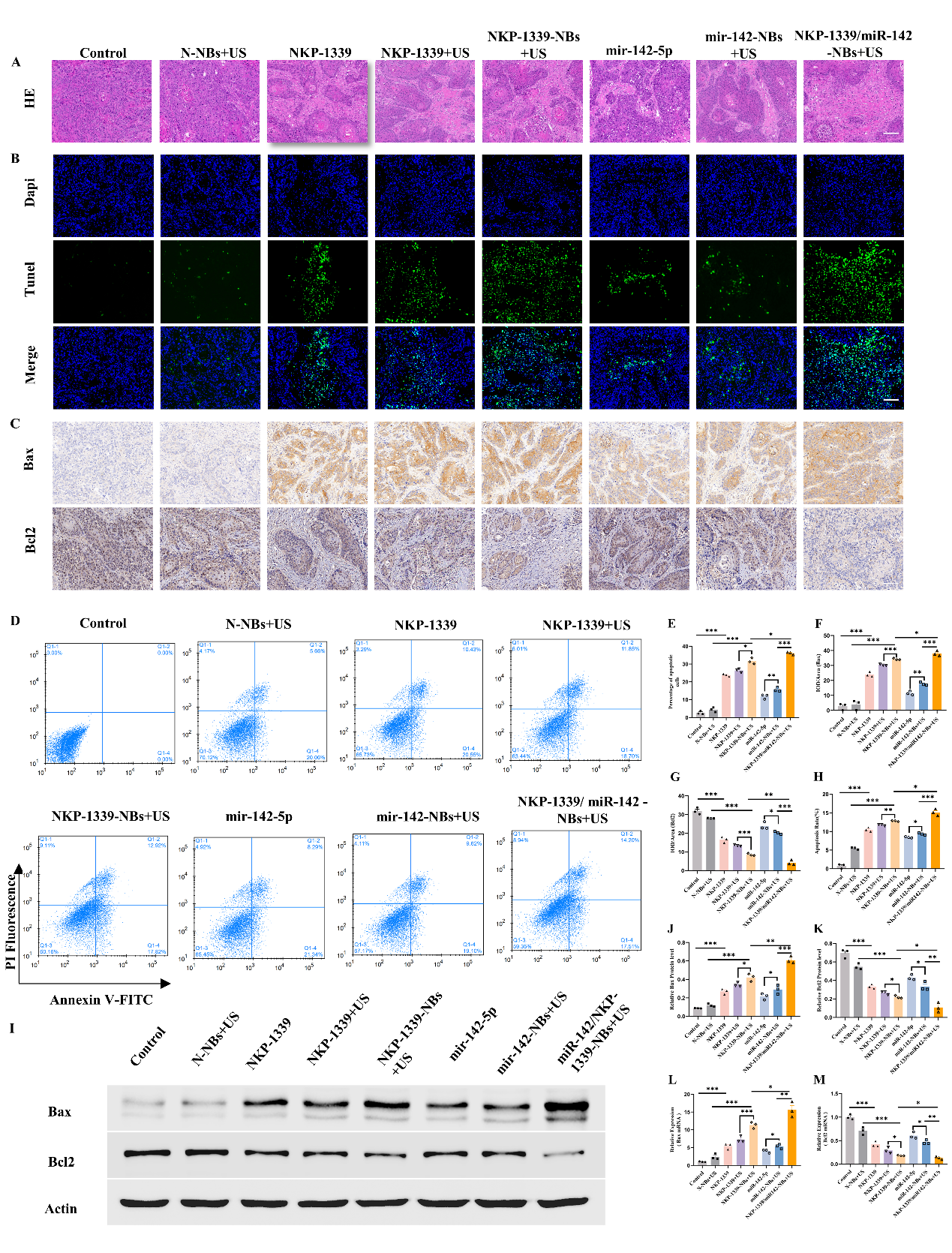
Fig.S1.** Analysis of antitumor activity of NKP-1339/miR-142-NBs combined with ultrasound treatment.(A) Hematoxylin and eosin (HE) staining to observe pathological changes in tumor tissue from each group (200× magnification, scale bar = 100 μm). (B and E) TUNEL assay to evaluate the apoptosis levels of tumor cells in each group, along with semi-quantitative analysis of apoptosis fluorescence intensity. (C, F, G) Immunohistochemical staining to detect the expression levels of Bax and Bcl-2 proteins (200× magnification, scale bar = 200 μm), with semi-quantitative analysis. (D and H) Flow cytometry to assess the proportion of apoptotic cells in each group and quantitative analysis. (I to K) Western blot (WB) analysis of Bax and Bcl-2 protein expression levels, accompanied by semi-quantitative analysis. (L to M) Quantitative PCR (qPCR) to assess the gene expression levels of Bax and Bcl-2. **P* < 0.05, ***P* < 0.01, ****P* < 0.001.


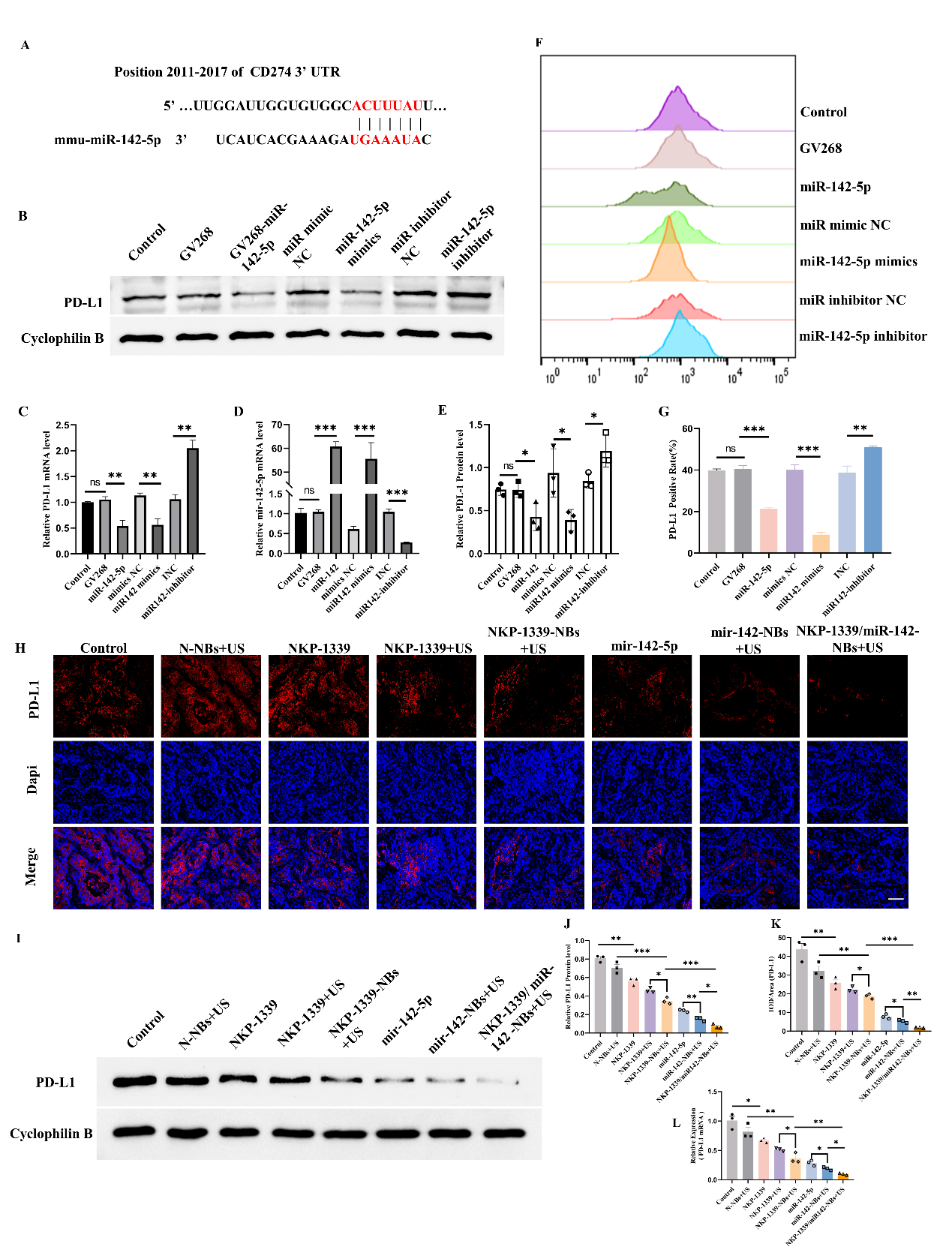
**Fig.S2.**MiR-142-5p targets PD-L1.(A)Bioinformatics analysis predicted the targeting relationship between miR-142-5p and PD-L1. (B) WB analysis of the effect of miR-142-5p on PD-L1 protein expression. (C) Semi-quantitative analysis of the Western blot band intensity for PD-L1. (D and E) Quantitative PCR (qPCR) to verify the changes in miR-142-5p and PD-L1 mRNA levels. (F to G) Flow cytometry to assess the expression levels of PD-L1 on the cell surface, along with quantitative analysis. (H and J) Immunofluorescence to detect PD-L1 protein levels *in vivo*, accompanied by semi-quantitative analysis. (I and K) WB to detect PD-L1 protein levels *in vivo*, with semi-quantitative analysis. (L) Reverse transcription-qPCR analysis of PD-L1 mRNA levels in mouse tumor tissues. **P* < 0.05, ***P* < 0.01, ****P* < 0.001.


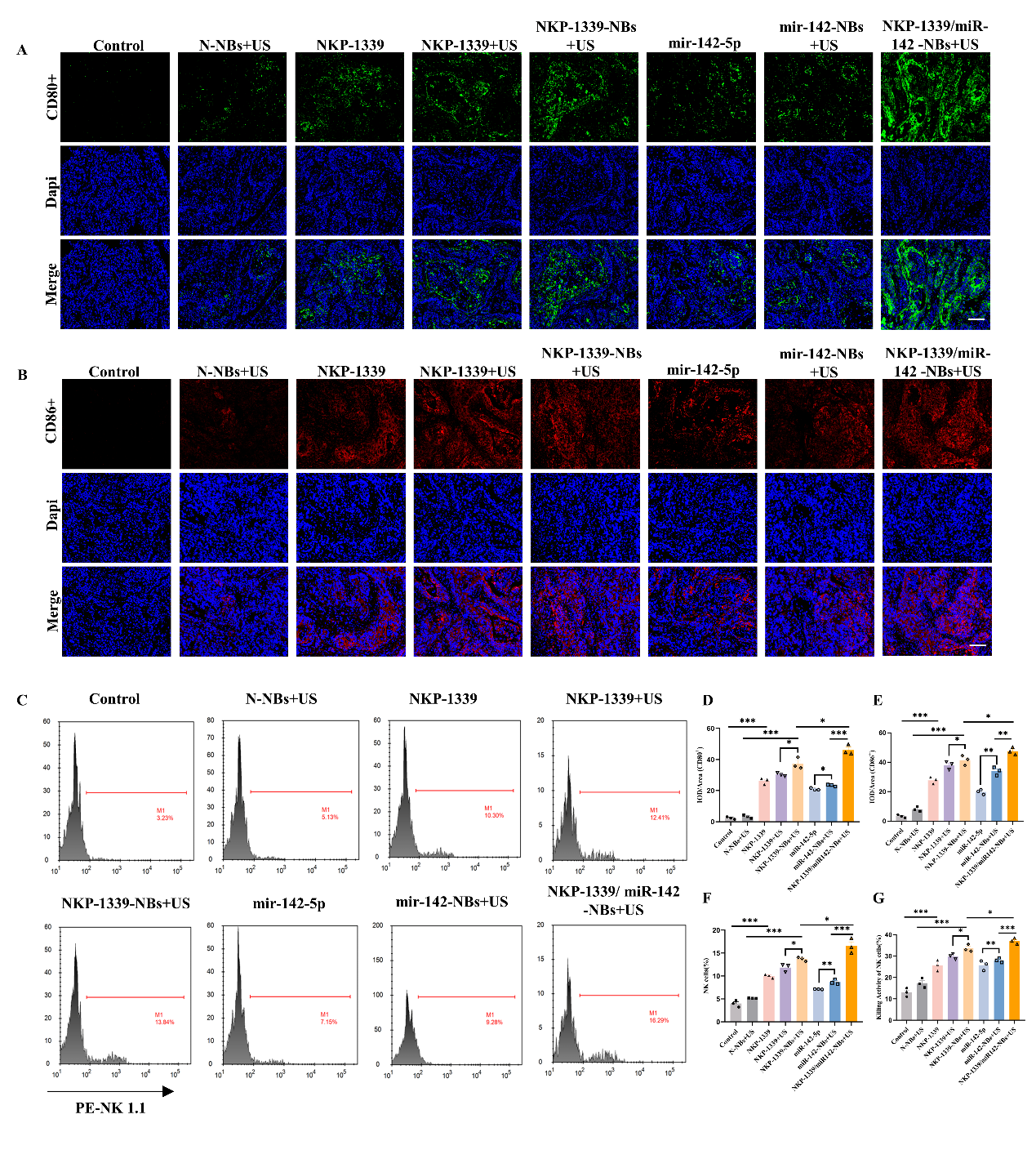
**Fig. S3.** Mechanism on immune response of NK cell and macrophage Induced by NKP-1339/miR-142-NBs combined with US treatment. (A and B) Immunofluorescence analysis of CD80 and CD86 protein expression levels in tumor tissues (200×, scale bar=100 μm). (C) Flow cytometry analysis of NK cell proportions in spleen. (D and E) Semi-quantification of the fluorescence intensity shown in (A and B). (F) Quantitative analysis of the proportion of NK cells in each group. (G) LDH release assay to measure NK cell cytotoxicity *in vitro*. **P* < 0.05, ***P* < 0.01, ****P* < 0.001.


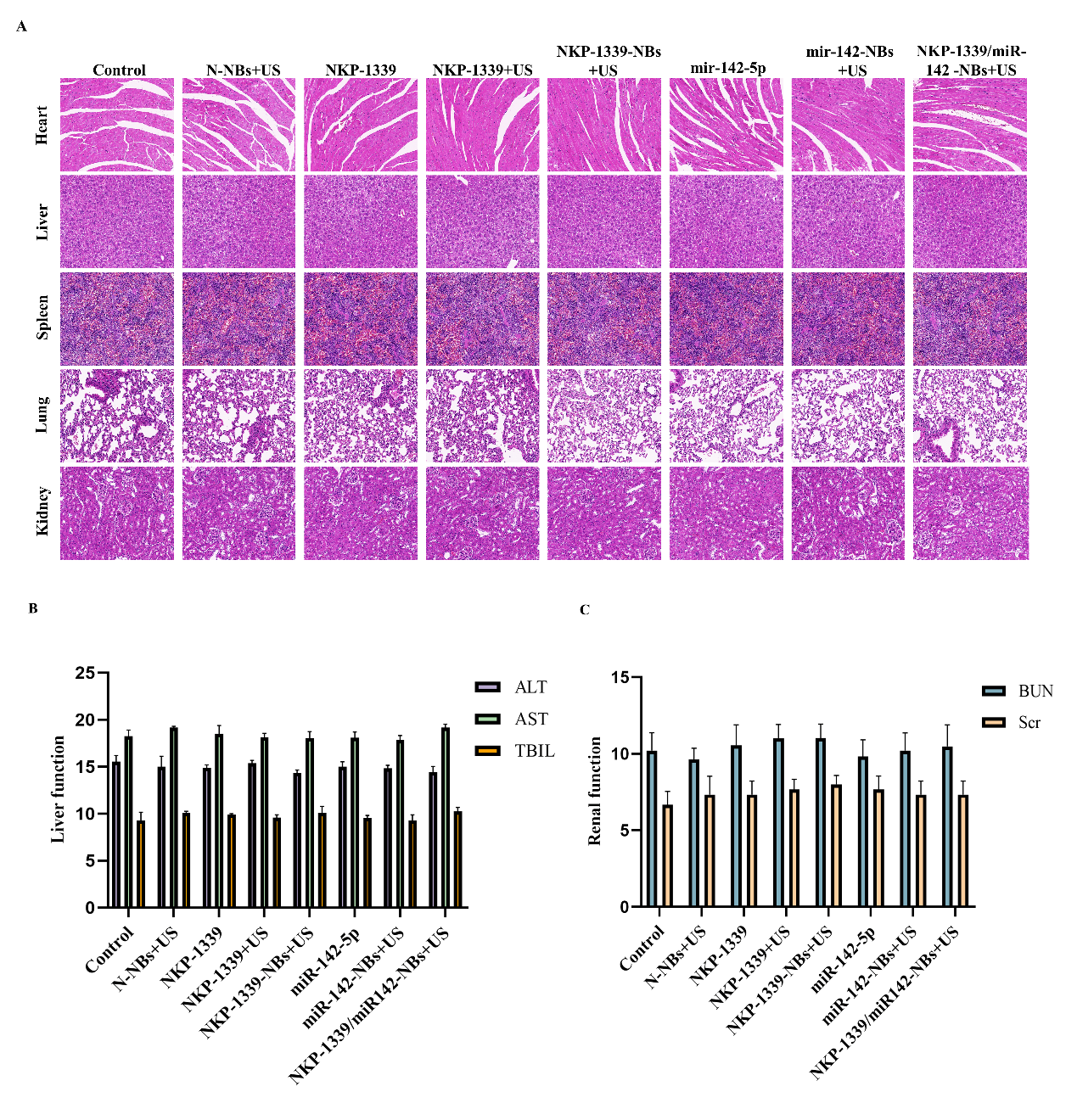
**Fig. S4.** *In vivo* safety evaluation of NKP-1339/miR-142-NBs combined with US therapy. (A) HE staining of major organs (heart, liver, spleen, lung, kidney) in each group of mice, observing histopathological changes (200×, scale bar =100 μm). (B and C) Quantitative analysis of liver and kidney indices in each group of mice.
